# VDLIN: A Deep Learning‐Based Platform for Methylcobalamin‐Inspired Immunomodulatory Compound Screening

**DOI:** 10.1002/advs.202413775

**Published:** 2025-10-27

**Authors:** Xuefei Guo, Yang Zhao, Xianle Rong, Chenxi Niu, Shiyu Hu, Qiang Liu, Fuping You

**Affiliations:** ^1^ Institute of Systems Biomedicine Department of Microbiology and Infectious Disease Center School of Basic Medical Sciences Beijing Key Laboratory of Tumor Systems Biology NHC Key Laboratory of Medical Immunology Peking University Health Science Center Beijing 100191 China; ^2^ Center of Basic Molecular Science (CBMS) Department of Chemistry Tsinghua University Beijing 100084 China; ^3^ Department of Pathology Xiangya Hospital School of Basic Medical Sciences Central South University Changsha 410013 China

**Keywords:** convolutional neural network, EGR1, inflammatory response, innate immunity, NF‐κB, TLR4, Vitamin B12 (VB12)

## Abstract

During the COVID‐19 pandemic, methylcobalamin (MCB), an active form of Vitamin B12 (VB12), showed therapeutic potential in mitigating the cytokine storm associated with SARS‐CoV‐2 infection. While MCB's significant anti‐inflammatory properties are confirmed, it is also observed that its treatment may impair macrophage‐mediated innate immune responses. Comprehensive RNA‐seq, ATAC‐seq, and CUT&Tag analyses revealed that MCB reduces inflammation and weakens innate immunity by limiting chromatin accessibility at NF‐κB and EGR1 binding sites, leading to decreased IFNB1 production and enhanced viral immune evasion. To address this challenge, a deep learning model, VDLIN (*Vitamin B12‐derived Deep Learning for Innate Immunity*), is developed to identify compounds capable of both suppressing inflammation and boosting innate immunity. As anticipated, VDLIN identified a novel compound, “Co7,” which retains MCB's strong anti‐inflammatory effects while also enhancing immune activation via the TLR4 signaling pathway. Co7 thus emerges as a promising therapeutic candidate, offering advantages over MCB by balancing anti‐inflammatory and immune‐stimulatory functions. Taken together, this study sheds light on the intricate interplay between chromatin dynamics and immune regulation, presenting new opportunities for therapeutic interventions in inflammatory diseases and SARS‐CoV‐2 infection.

## Graphical abstract

Using the convolutional neural network model VDLIN, Co7 is identified as a promising therapeutic candidate. Co7 demonstrates distinct advantages over MCB by effectively balancing anti‐inflammatory and immune‐stimulatory functions, making it a potential novel approach for immune modulation.

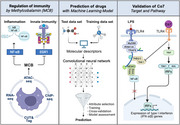

## 1 Introduction

A hyperactive immune response, commonly referred to as a cytokine storm, can be initiated by SARS‐CoV‐2 infection and is characterized by the excessive release of pro‐inflammatory cytokines, including IL‐6, IL‐1β, and TNF‐α.^[^ ^1^, ^2^, ^3^, ^4^, ^5^, ^6^ ^]^ This dysregulated response, primarily driven by the innate immune system, triggers a cascade of immune cell recruitment and signaling pathways aimed at clearing the virus.^[^ ^7^, ^8^, ^9^ ^]^ However, unchecked cytokine production results in widespread systemic inflammation, severe tissue damage, and increased vascular permeability.^[^ ^10^ ^]^ In severe cases of COVID‐19, this uncontrolled inflammatory response culminates in acute respiratory distress syndrome (ARDS), multi‐organ failure, and, frequently, death.^[^ ^11^ ^]^ The persistent elevation of cytokines amplifies the immune response, creating a vicious cycle of inflammation and tissue destruction.^[^ ^12^ ^]^ A comprehensive understanding of the mechanisms underlying the cytokine storm is essential for developing effective interventions to mitigate mortality associated with COVID‐19. Additionally, SARS‐CoV‐2 undermines the antiviral defenses of the host by suppressing type I interferon signaling and facilitating immune evasion.^[^ ^13^, ^14^ ^]^ These insights highlight the urgent need for therapeutic strategies capable of modulating immune responses to reduce inflammation while preserving antiviral immunity.

Methylcobalamin (MCB), the active form of Vitamin B12, serves as a crucial cofactor in the methionine cycle, facilitating the synthesis of S‐adenosylmethionine (SAM), the primary methyl donor in DNA methylation processes.^[^ ^15^, ^16^, ^17^ ^]^ This cycle begins with the conversion of homocysteine to methionine, catalyzed by methionine synthase, followed by the transformation of methionine into SAM. SAM donates methyl groups to DNA, particularly at CpG dinucleotides, through the action of DNA methyltransferases (DNMTs), resulting in the formation of 5‐methylcytosine, an epigenetic marker typically associated with gene repression. By maintaining appropriate methylation levels, MCB supports essential DNA methylation patterns critical for development, genomic imprinting, and suppressing transposable elements.^[^ ^18^, ^19^, ^20^ ^]^ Disruptions in these methylation patterns have been linked to various diseases, including cancer, neurodegenerative disorders, and autoimmune conditions, highlighting MCB's role in genomic stability and gene regulation.^[^ ^21^, ^22^ ^]^ Furthermore, MCB demonstrates potent anti‐inflammatory and neuroprotective effects by modulating key pro‐inflammatory cytokines, such as TNF‐α, IL‐1β, and IL‐6.^[^ ^23^ ^]^ MCB has also been utilized to mitigate hyperinflammatory responses, including the cytokine storm associated with COVID‐19, potentially reducing disease severity and improving clinical outcomes.^[^ ^24^, ^25^, ^26^ ^]^ However, the precise molecular mechanisms by which MCB exerts its anti‐inflammatory effects, as well as its potential to enhance the antiviral type I interferon response, warrant further investigation.

In recent years, phosphines‐nitrogen‐phosphines (PNP) pincer ligands have garnered significant attention due to their unique potential in the fields of catalysis and coordination chemistry. The defining structural feature of PNP ligands lies in their central nitrogen atom, which, in synergy with two flanking phosphine groups, enables robust σ‐donor and π‐acceptor properties. This configuration allows PNP ligands to form stable coordination complexes with various metal ions. Their distinctive geometric and electronic characteristics endow them with exceptional catalytic activity and selectivity, making them up‐and‐coming candidates for coordination and catalytic chemistry applications. Despite the substantial progress made in understanding and applying PNP ligands within these domains, their potential biological applications—particularly in modulating immune responses, cancer therapy, and antiviral strategies—remain largely unexplored. Therefore, an in‐depth investigation into the immunomodulatory roles of PNP ligands may offer valuable theoretical insights and practical support for developing novel therapeutic approaches.

The integration of machine learning (ML) algorithms into drug screening has revolutionized pharmaceutical research, accelerating the identification and development of therapeutic agents.^[^ ^27^, ^28^, ^29^ ^]^ Advanced ML models, such as deep neural networks, convolutional neural networks (CNN), and support vector machines (SVM), are adept at analyzing complex biological datasets to predict drug efficacy and safety, thereby streamlining drug discovery and reducing associated costs.^[^ ^30^, ^31^ ^]^ These approaches also facilitate drug repurposing by leveraging extensive pharmaceutical databases to identify novel therapeutic applications for existing compounds.^[^ ^32^ ^]^ Furthermore, deep learning has facilitated drug discovery by leveraging open‐source frameworks or libraries such as TensorFlow and Keras, which enable the efficient construction of complex models and the scalable analysis of large‐scale biomedical datasets.^[^ ^33^ ^]^ This methodology uncovers intricate patterns within large‐scale datasets, improving various aspects of drug discovery, including de novo design, retrosynthetic analysis, and molecular property prediction.^[^ ^34^ ^]^ In this study, we demonstrated that while MCB effectively inhibited excessive inflammatory responses, it also diminished the host's antiviral innate immune response. To address this dual regulatory challenge, we developed the VDLIN model (*Vitamin B12‐derived Deep Learning for Innate Immunity*), a CNN‐based framework designed to capture transcriptomic signatures associated with MCB‐mediated immune modulation. This model was utilized to screen for the small‐molecule compound Co7, which simultaneously suppresses inflammation and enhances the interferon (IFN) response. We further investigated the targets and molecular mechanisms of Co7 to elucidate its therapeutic potential.

## 2 Results

### 2.1 MCB Inhibits the Inflammatory Response Induced by LPS

**Figure 1:**
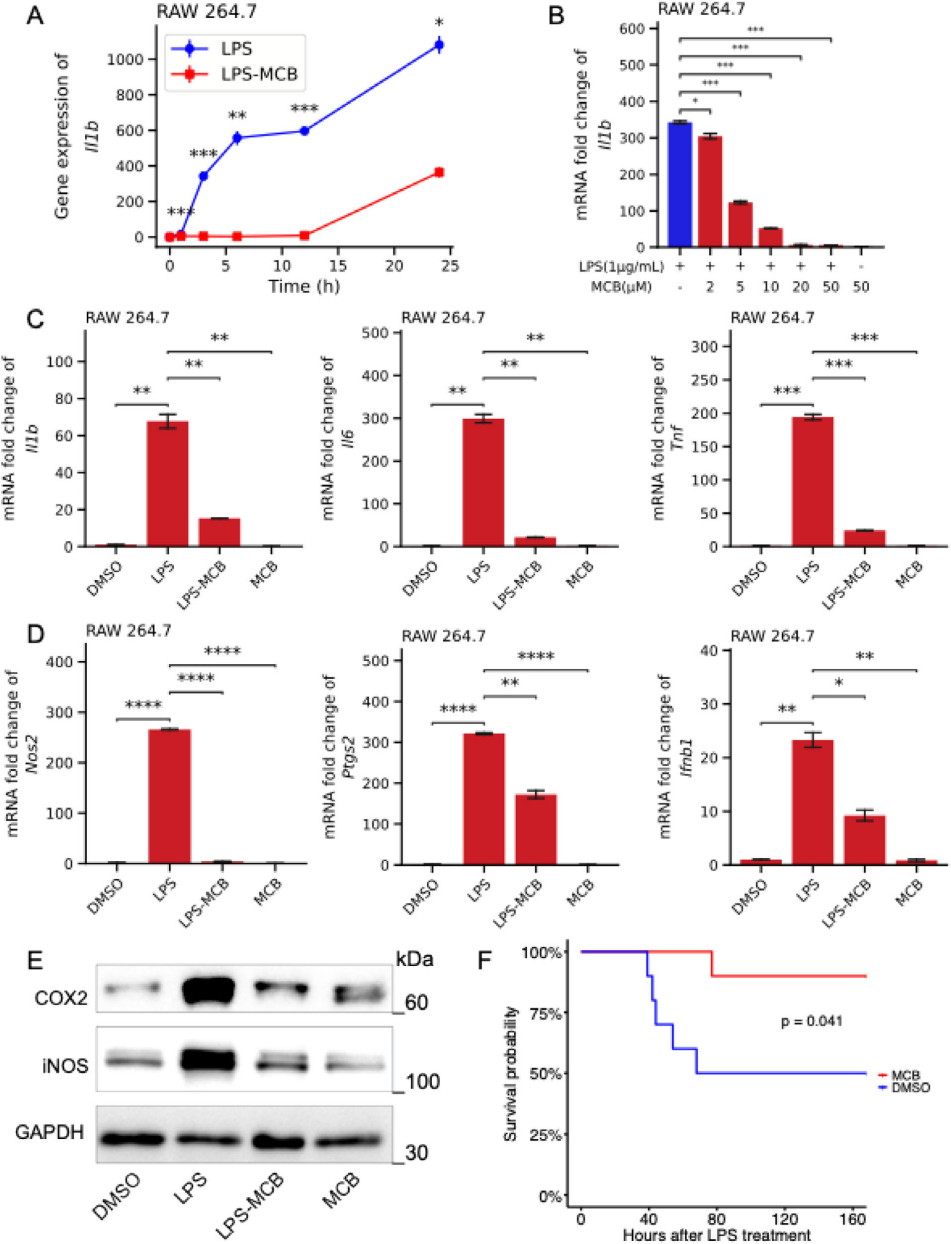
Methylcobalamin (MCB) suppressed LPS‐induced inflammatory responses in RAW 264.7 cells. A) MCB (50 µmol/L) treatment suppressed *Il1b* expression induced by LPS (1 µg/mL) at multiple time points, including 0, 1, 3, 6, 9, 12, and 24 h, indicating a time‐dependent inhibitory effect. B) MCB reduced LPS (1 µg/mL)‐induced *Il1b* expression in a concentration‐dependent manner, with significant suppression observed at concentrations of 2, 5, 10, 20, and 50 µmol/L after 12 h of treatment, as determined by RT‐qPCR analysis. C‐D) MCB markedly downregulated the expression of pro‐inflammatory cytokines (*Il1b*, *Il6*, and *Tnf*), two key inflammatory enzymes (*Nos2* and *Ptgs2*), and the type I interferon *Ifnb1* following 12 h of LPS stimulation and treatment with 20 µmol/L MCB, as determined by RT‐qPCR analysis. E) Immunoblot analysis of *Nos2* and *Ptgs2* protein expression levels following 12‐h treatment with DMSO, LPS, LPS‐MCB, or MCB, with GAPDH as the internal loading control. F) MCB significantly reduced the mortality rate in mice (n = 10 per group) following LPS challenge (20 mg/kg). RT‐qPCR data were presented as means ± SEM from three independent experiments. Statistical significance was determined by one‐way ANOVA with Bonferroni's multiple comparisons test (B–D), paired‐samples t‐test (A), or the log‐rank test (F). *P < 0.05, **P < 0.01, and ***P < 0.001.

We initially assessed the anti‐inflammatory effects of methyl‐vitamin B12 (methylcobalamin, MCB) using the murine macrophage cell line RAW 264.7 stimulated with lipopolysaccharide (LPS). MCB treatment led to a significant, time‐dependent reduction in the mRNA levels of key pro‐inflammatory genes, including *Il1b*, *Il6*, and *Nos2*, with maximal suppression observed at 12 h post‐LPS stimulation (**Figures** **1**A and S1B, Supporting Information). Furthermore, MCB exhibited dose‐dependent inhibitory effects on *Il1b* and *Il6* expression across a concentration range of 2–50 µM, with near‐maximal efficacy achieved at 20 µM (Figure 1B and Figure S1A, Supporting Information). Based on these results, 20 µM and 12 h post‐treatment were selected as optimal conditions for subsequent mechanistic investigations.

Expanded transcript analysis revealed that MCB broadly suppressed pro‐inflammatory cytokines and chemokines, including *Tnf*, *Ptgs2*, *Ccl3*, *Ccl4*, *Lta*, and *Ltb* (Figure 1C,D and Figure S1C, Supporting Information). Notably, MCB also attenuated innate immune signaling by downregulating type I interferon (*Ifnb1*) and interferon‐stimulated genes (ISGs) such as *Cxcl10*, *Cxcl11*, *Ifit1*, and *Isg15* (Figure 1D and Figure S1A,D, Supporting Information). Consistent with transcriptional changes, protein‐level analysis demonstrated substantial reductions in inducible nitric oxide synthase (iNOS) and cyclooxygenase‐2 (COX2), both critical effectors in inflammatory cascades (Figure 1E). Importantly, in an in vivo LPS‐induced sepsis model, MCB significantly improved survival rates (Figure 1F). Collectively, these findings demonstrate that MCB exerts potent anti‐inflammatory effects both in vitro and in vivo, primarily through modulation of key inflammatory mediators and pathways.

To elucidate the molecular mechanisms underlying MCB's anti‐inflammatory activity, we performed bulk RNA sequencing (RNA‐seq) on RAW 264.7 cells treated with LPS or LPS plus MCB. Principal component analysis (PCA) revealed a distinct shift in the global transcriptomic profile upon MCB treatment, indicating a substantial reversal of LPS‐induced transcriptional changes (Figure S2A, Supporting Information). Differential expression analysis identified a set of genes significantly downregulated by MCB, many of which are classical inflammatory mediators, including *Tnf*, *Il6*, *Ccl4*, *Csf3*, *Il27*, and *Saa3* (Figure S2B, Supporting Information). Conversely, MCB upregulated genes associated with membrane transport, autophagy, metabolism, and oxidative stress regulation, such as *Acp5*, *Dusp14*, *Ulk1*, *Morn4*, *Sesn3*, *Slc37a2*, and *Slc5a3* (Figure S2B, Supporting Information). These data suggest that MCB not only suppresses pro‐inflammatory gene expression but also promotes cellular homeostasis and stress‐adaptive processes, highlighting its dual role in immunomodulation and metabolic regulation.

### 2.2 MCB Inhibits the Inflammatory Response Through NFKB

To further explore the biological processes regulated by MCB, we performed GO and KEGG enrichment analyses of the DEGs. Genes upregulated in the LPS‐MCB group were enriched in pathways related to lipid metabolism, apoptosis, mitophagy, autophagy, and protein tyrosine phosphatase activity (Figure S2C, Supporting Information). In contrast, downregulated genes were significantly enriched in pro‐inflammatory pathways, including TNF, NF‐κB, chemokine, Toll‐like receptor, JAK–STAT signaling, and cellular responses to IL‐1 and TNF (Figures S2D and S3A, Supporting Information). GSEA further confirmed that hallmark pathways such as inflammatory response, IL6‐JAK‐STAT3‐signaling, and TNFA signaling via NFKB were suppressed in the LPS‐MCB group (Figure S2E and S3B,C, Supporting Information). In contrast, metabolic pathways including HEME metabolism and BILE ACID metabolism were activated (Figure S3D,E, Supporting Information).

**Figure 2:**
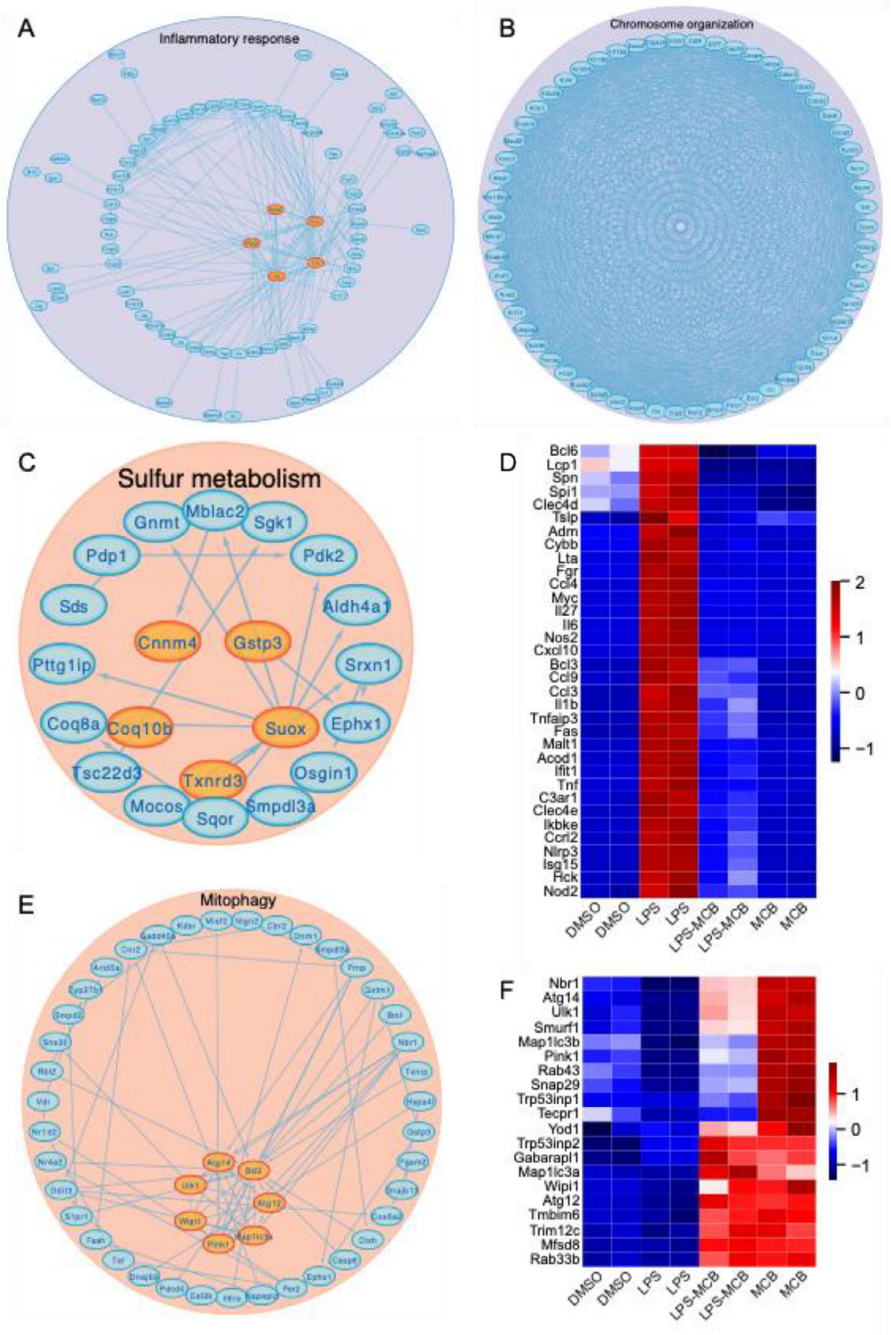
Protein–protein interaction (PPI) network analysis of differentially expressed genes (DEGs) identified from RNA‐seq data comparing LPS‐MCB and LPS treatments for 12 h in RAW 264.7 cells. A) Downregulated DEGs by MCB in the PPI network were significantly enriched in the inflammatory response, with hub genes including *Il1b*, *Il6*, *Tnf*, *Nos2*, and *Ptgs2*. B) Downregulated DEGs by MCB in the PPI network were significantly enriched in processes related to chromosome organization. C) Upregulated DEGs by MCB in the PPI network were significantly enriched in sulfur metabolism, containing hub genes such as *Coq10b*, *Cnnm4*, *Gstp3*, *Suox*, and *Txnrd3*. D) Heatmap illustrating the expression of upregulated genes in the LPS group compared to other groups, specifically those involved in the inflammatory response pathway. E) Upregulated DEGs by MCB in the PPI network were significantly enriched in the mitophagy pathway, including hub genes such as *Atg12*, *Atg14*, *Ulk1*, *Wipi1*, *Pink1*, and *Map1lc3a*. F) Heatmap depicting the upregulated DEGs induced by MCB associated with the mitophagy pathway.

We constructed a protein–protein interaction (PPI) network to identify functional interactions. Downregulated DEGs were primarily involved in inflammation, chromosome organization, DNA replication, and cell cycle regulation (**Figures** **2**A,B and S4A,B, Supporting Information). A heatmap of representative genes, including *Il1b*, *Il6*, *Il17*, *Ccl3*, *Ccl9*, *Acod1*, and *Tnf*, showed consistent downregulation upon MCB treatment (Figure 2D), with expression levels detailed in Figure S4D (Supporting Information). Conversely, upregulated genes were enriched in sulfur metabolism, mitophagy, and lipid metabolism (Figures 2C,E and S4C, Supporting Information). Notably, mitophagy‐related genes such as *Atg12*, *Atg14*, *Map1lc3a*, *Nbr1*, *Pink1*, and *Ulk1* were markedly induced by MCB in both LPS and non‐LPS contexts (Figures 2F and S4E, Supporting Information), suggesting that mitophagy may contribute to its anti‐inflammatory action.

**Figure 3:**
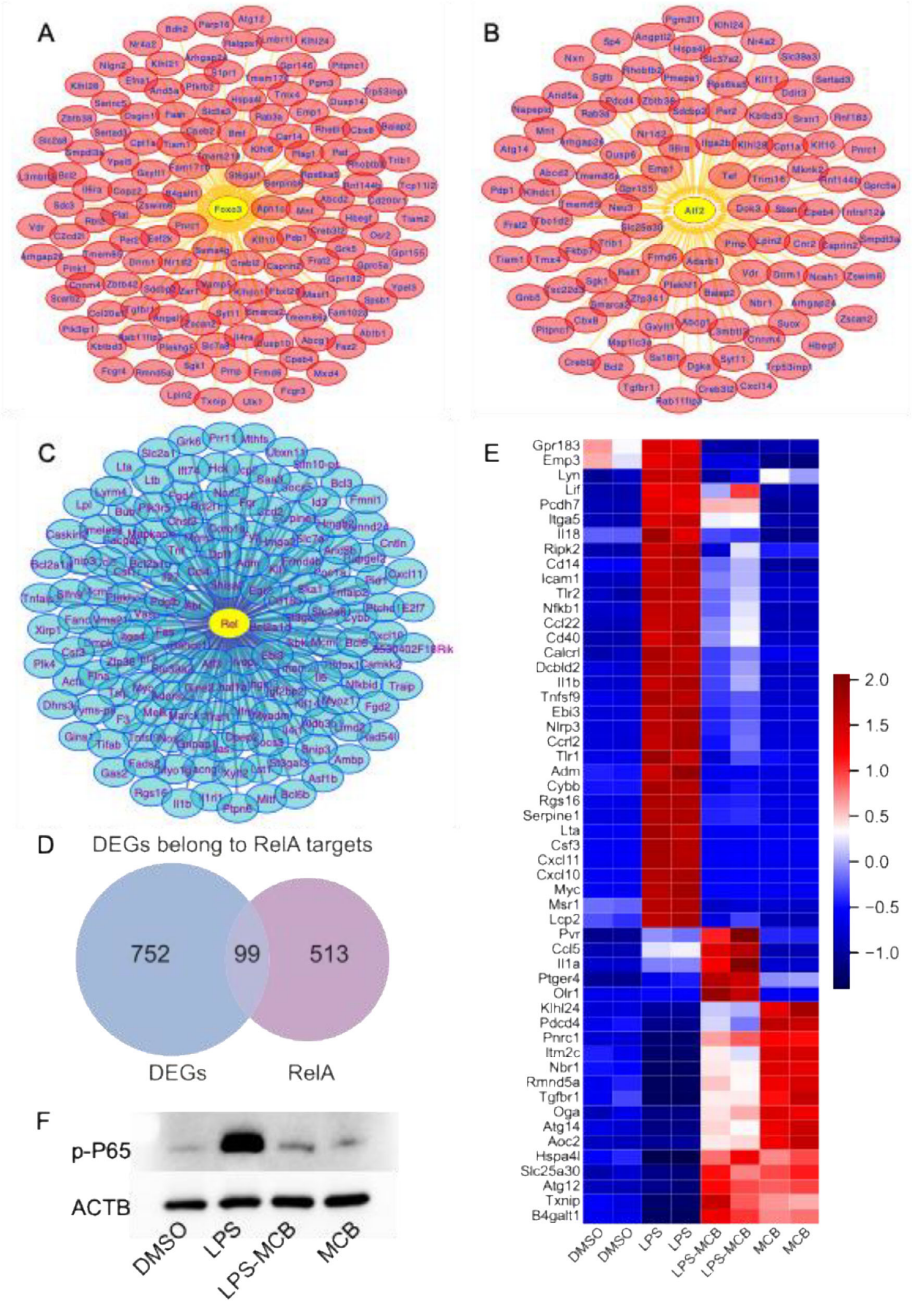
MCB attenuates the LPS‐induced inflammatory response by modulating the NF‐κB signaling pathway in RAW 264.7 cells following 12‐h treatment. A,B) Transcription factor enrichment analysis (TFEA) of upregulated differentially expressed genes (DEGs) in the LPS‐MCB group versus the LPS group identified *Foxo3* and *Atf2* as key candidate regulators. Yellow nodes indicate enriched transcription factors, while red nodes represent upregulated DEGs in the LPS‐MCB group relative to the LPS group. C) TFEA of downregulated DEGs in the LPS‐MCB group relative to the LPS group identified *Rel* as a potential regulatory factor. Yellow nodes represent enriched transcription factors, while blue nodes indicate DEGs downregulated following MCB treatment. D) The Venn diagram illustrates 99 DEGs between the LPS‐MCB and LPS groups that overlap with known *RelA* target genes. E) Heatmap illustrating the expression of target genes regulated by *RelA* and *Foxo3* in response to treatment conditions. The color scale represents the relative expression levels of each gene, with red indicating upregulation and blue indicating downregulation. F) Western blot analysis showing that MCB significantly inhibited LPS‐induced phosphorylation of the P65 transcription factor upon 12‐h treatment in RAW 264.7 cells. ACTB was used as the loading control.

Transcription factor (TF) enrichment was conducted using iRegulon to identify upstream regulators. Upregulated genes were associated with TFs such as Foxo3, Atf2, and Foxn3 (**Figures** **3**A,B and S5A, Supporting Information), whereas downregulated genes were linked to Rel, E2f1, Stat1, Nfya, and Sfpi1 (Figures 3C and S5B–E, Supporting Information). Notably, 99 downregulated DEGs were predicted to be targets of RelA (Figure 3D,E), suggesting the suppression of NF‐κB signaling. This was validated by immunoblotting, which showed that MCB markedly inhibited LPS‐induced phosphorylation of NF‐κB p65 (Figure 3F). Together, these results suggest that MCB primarily attenuates inflammatory responses by inhibiting the NF‐κB pathway.

### 2.3 MCB Regulates Chromatin Accessibility to Reprogram the LPS Response

**Figure 4:**
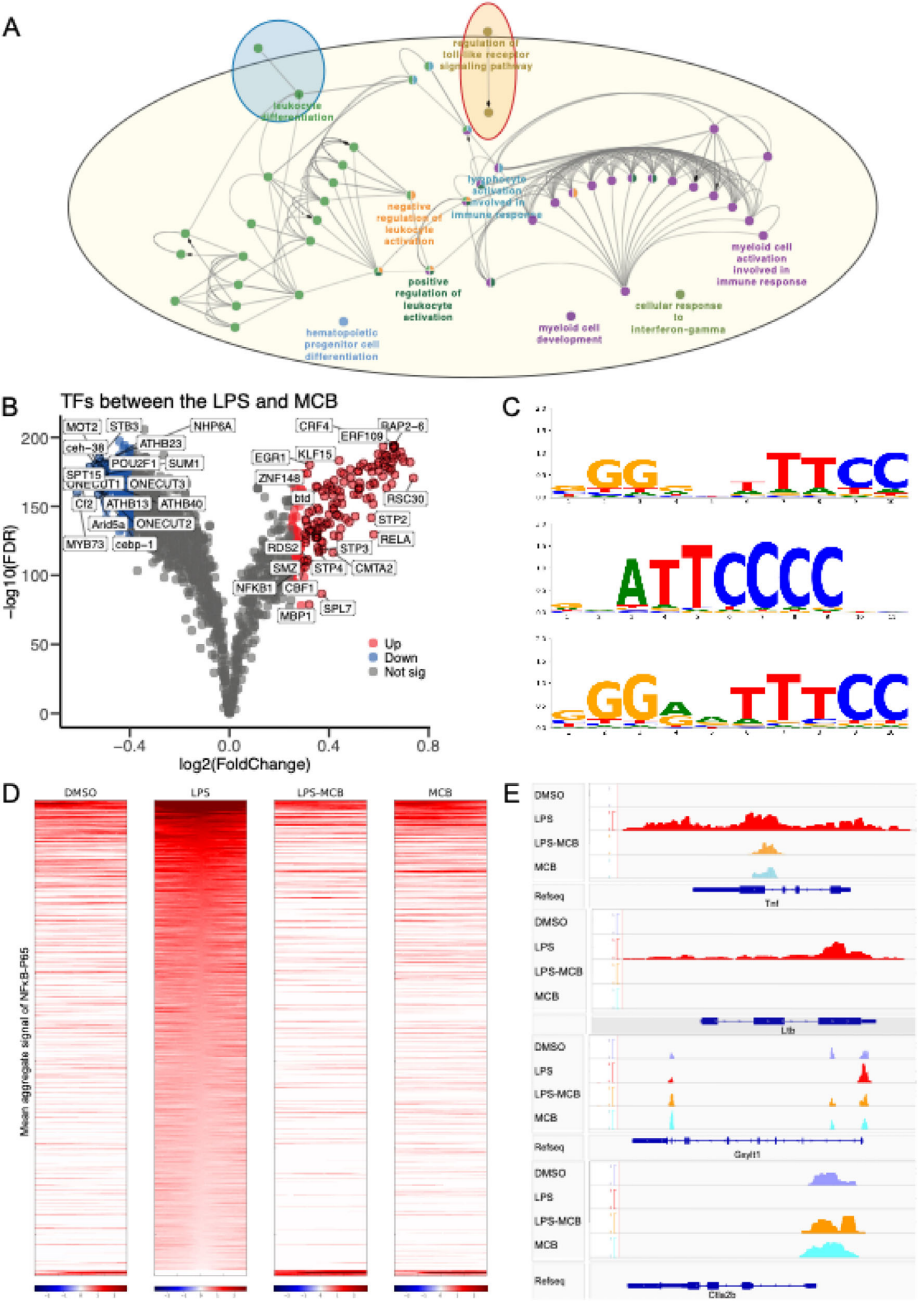
MCB reduces LPS‐induced chromatin accessibility at NF‐κB (P65) binding sites in RAW 264.7 cells following 12 h of treatment. A) Gene Ontology (GO) annotation of differentially accessible regions (DARs) associated with genes in the LPS group relative to other groups, highlighting enrichment in biological processes such as myeloid cell development, cellular response to interferon‐gamma, positive regulation of leukocyte activation, and regulation of toll‐like receptor signaling pathways. B) Volcano plot showing the enrichment of potential transcription factors (PTFs) between the LPS and MCB groups, with fold change transformed using “log2”. Significantly enriched PTFs in the LPS group are indicated by red dots, while significantly enriched PTFs in the MCB group are shown in blue. C) NF‐κB‐P65 binding motifs obtained from the JASPAR database. D) Mean aggregate binding signal of NF‐κB‐P65 across the DMSO, LPS, LPS‐MCB, and MCB groups. E) Visualization of chromatin accessibility peak tracks for the DMSO, LPS, LPS‐MCB, and MCB groups using IGV software.

To investigate whether MCB attenuates LPS‐induced NF‐κB activation through epigenetic mechanisms, we performed ATAC‐seq on RAW 264.7 cells treated with LPS and/or MCB. As shown in Figures S6A–C and S7A (Supporting Information), the LPS group exhibited 33724 accessible peaks, markedly higher than those in the LPS‐MCB (28373) and MCB (23017) groups. Heatmaps of peak distribution were presented in Figure S6D–F (Supporting Information). Differential accessibility analysis identified 7097 differentially accessible regions (DARs) between the LPS and other groups (Figure S7B, Supporting Information). Functional annotation via GREAT and visualization with Cytoscape (v3.10.2) revealed that LPS‐induced DARs were enriched in immune‐related processes, including immune response, antigen presentation, NF‐κB regulation, and viral defense (Figure S7C, Supporting Information). ChIPseeker analysis showed that LPS‐induced DARs were more frequently located in promoter regions and less in intergenic regions compared to the LPS‐MCB and MCB groups (Figure S7D, Supporting Information). Moreover, the LPS group exhibited a higher density of transcription factor (TF) binding sites within 3 kb of TSSs, indicative of elevated transcriptional activity (Figure S7E, Supporting Information). ClueGO analysis of genes near LPS versus LPS‐MCB DARs further confirmed enrichment in Toll‐like receptor signaling, leukocyte differentiation, and interferon‐γ response (**Figure** **4**A).

To identify upstream regulatory factors, we performed TF footprinting using TOBIAS (v0.17.0). *Rela* and *Nfkb1* showed significantly increased binding activity in the LPS group (Figure 4B), with the *Rela* motif illustrated in Figure 4C. Heatmaps revealed elevated NF‐κB p65 occupancy in the LPS group compared to DMSO, LPS‐MCB, and MCB groups (Figure 4D). MCB markedly suppressed binding of pro‐inflammatory TFs, including *Nfkb1*, *Nfkb2*, *Irf3*, *Fosl1*, *Jund*, and *Stat1* (Figure S8A,B, Supporting Information), highlighting its broad anti‐inflammatory action. Conversely, TFs involved in chromatin regulation, such as *Cebp1*, *Sum1*, *Arid5a*, and *Spt15*, showed increased binding in the MCB group (Figure 4B, Figure S8C,D, Supporting Information). Integrating ATAC‐seq and RNA‐seq data, we identified 258 genes that were both differentially expressed and proximal to DARs (Figure S9A, Supporting Information). IGV visualization showed greater chromatin accessibility at inflammatory loci (*Tnf*, *Lta*, *Ltb*, *Vma21*, *Acod1*, *Hmgb1*) in the LPS group, while genes such as *Ctla2b* were more accessible in the LPS‐MCB and MCB groups (Figure 4E, Figure S9B, Supporting Information). FPKM values were consistent with chromatin accessibility profiles (Figure S9C, Supporting Information). Notably, MCB altered both chromatin accessibility and expression of several key TFs and histone‐coding genes, including *Atf3*, *Irf3*, *H4c9*, *H2ac11*, and *H2bc11* (Figure S9B,C, Supporting Information), underscoring a chromatin‐level mechanism for its immunomodulatory effects.

### 2.4 MCB Decreases the Recruitment of EGR1 to Promoters of ISGs

**Figure 5:**
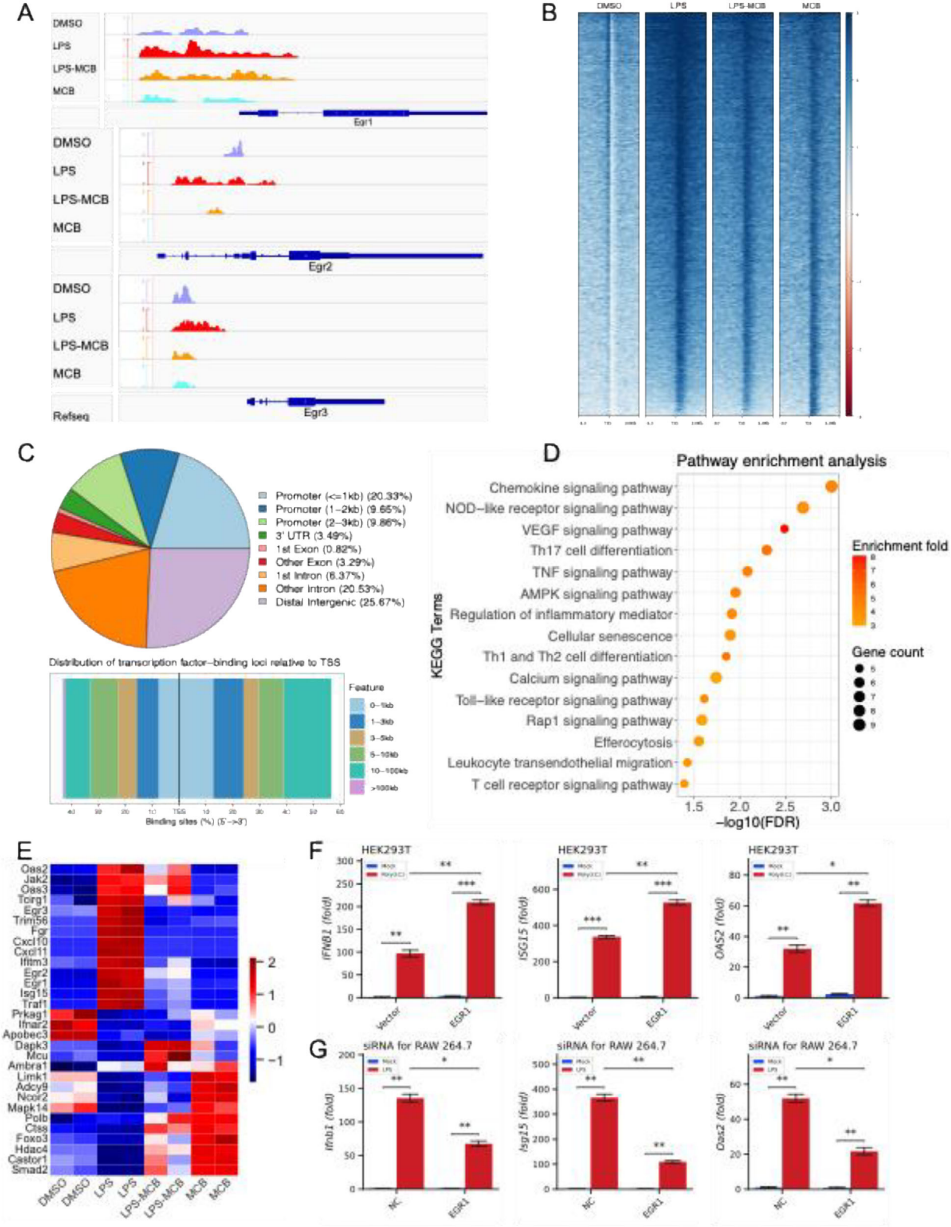
LPS‐induced *Egr1* plays a crucial role in the innate immune response. A) Chromatin accessibility at *Egr1*, *Egr2*, and *Egr3* loci, which was markedly increased upon LPS stimulation, was significantly reduced following 12‐h MCB treatment in RAW 264.7 cells. B) Mean aggregate binding signal of *Egr1* across the DMSO, LPS, LPS‐MCB, and MCB groups, as assessed by CUT&Tag in RAW 264.7 cells following 12‐h treatment. C) Statistics and distribution of transcription factor‐binding loci relative to transcription start sites (TSS) of differentially accessible regions (DARs) detected by CUT&Tag in the LPS group compared to the MCB group. D) Annotation of these DARs detected by CUT&Tag revealed significant enrichment in KEGG pathways, including the NOD‐like receptor signaling pathway, VEGF signaling pathway, TNF signaling pathway, and Toll‐like receptor signaling pathway, when comparing the LPS and MCB groups. E) Heatmap representing the expression of potential target genes of *Egr1*. F) Overexpression of *Egr1* in HEK293T cells enhanced the expression of *IFNB1*, *ISG15*, and *OAS2* upon Poly(I:C) stimulation in HEK293T cells. G) Knockdown of *Egr1* using shRNA in RAW 264.7 cells reduced the expression of *Ifnb1*, *Isg15*, and *Oas2* induced by LPS in RAW 264.7 cells. RT‐qPCR data were presented as means ± SEM from three independent experiments. Statistical significance was determined using a paired‐samples t‐test (F‐G). *P < 0.05, **P < 0.01, and ***P < 0.001.

Three zinc finger transcription factors—*Egr1*, *Egr2*, and *Egr3*—exhibited significantly higher chromatin accessibility and expression in the LPS group compared to other groups (**Figures** **5**A and S10A, Supporting Information). Among them, *Egr1* showed the most pronounced increase and was substantially downregulated following MCB treatment. Motif footprinting revealed a markedly stronger *Egr1* binding signal in the LPS group (Figure S10B,C, Supporting Information), consistent with its elevated expression. To further clarify *Egr1*’s regulatory role, we performed CUT&Tag analysis, which confirmed increased *Egr1* occupancy in the LPS group (Figure 5B). These DARs were predominantly located near transcription start sites (TSSs) and enriched in promoters, 3′UTRs, and introns, particularly within ±3 kb of TSSs (Figures 5C and S10D–F, Supporting Information). KEGG pathway enrichment analysis of *Egr1*‐bound regions highlighted key innate immune pathways, including NOD‐like receptor signaling, TNF signaling, and Toll‐like receptor pathways (Figure 5D), underscoring *Egr1*’s central role in regulating inflammatory genes. Expression analysis of *Egr1* target genes revealed that multiple ISGs—including *Jak2*, *Oas2*, *Oas3*, *Cxcl10*, *Isg15*, and *Ifitm3*—were downregulated by MCB (Figure 5E).

To functionally validate *Egr1*’s involvement, we overexpressed *Egr1* in HEK293T cells, which led to a significant upregulation of *Ifnb1*, *Isg15*, and *Oas2* (Figure 5F). Conversely, shRNA‐mediated knockdown of *Egr1* in RAW 264.7 cells markedly attenuated LPS‐induced expression of these same genes (Figure 5G). These results are consistent with prior studies that have implicated *Egr1* in antiviral defense.^[^ ^35^ ^]^ Collectively, our findings demonstrate that MCB suppresses inflammation via the NF‐κB pathway and dampens *Egr1*‐mediated innate immune responses. While the immunosuppressive effect of MCB may benefit individuals with inflammatory diseases, it could also compromise their antiviral immunity, potentially promoting immune evasion.

### 2.5 A Better MCB Was Identified Based on Machine‐Learning Methods

**Figure 6:**
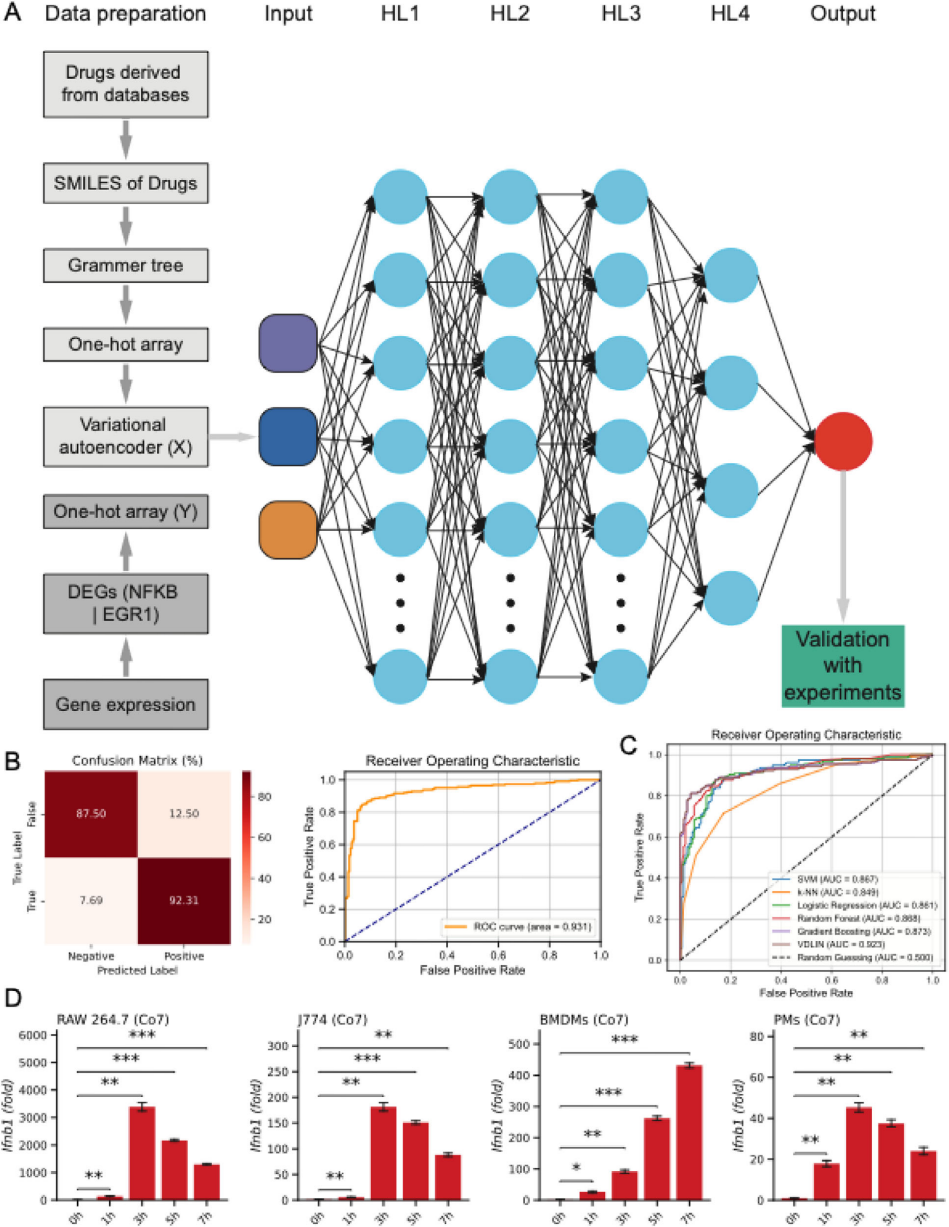
Co7, an optimized derivative of MCB, was identified through screening with the convolutional neural network (CNN) model VALIN. A) Diagram depicting data preparation and the construction of the CNN model VALIN, designed to screen compounds that inhibit the inflammatory response mediated by NFKB and enhance the innate immune response through *Egr1*. B) Evaluation of VALIN's performance using a confusion matrix, receiver operating characteristic (ROC) curve, and area under the curve (AUC). C) ROC curve comparison of multiple machine learning models evaluated using the VALID framework. Models include Support Vector Machine (SVM), k‐Nearest Neighbors (kNN), Logistic Regression, Random Forest, and Gradient Boosting. D) Co7 (50 µmol/L) significantly induced the expression of *Ifnb1* over time in RAW 264.7, J774, BMDMs, and PMs, respectively. RT‐qPCR data were presented as means ± SEM from three independent experiments. Statistical significance was determined using one‐way ANOVA with Bonferroni's multiple comparisons test (C). *P < 0.05, **P < 0.01, and ***P < 0.001.

To identify pharmacological agents that simultaneously suppress *NF‐κB*‐driven inflammatory signatures and enhance *Egr1*‐mediated innate immune responses, we developed VDLIN, a convolutional neural network (CNN) model inspired by the immunomodulatory properties of vitamin B12 (VB12) (**Figure** **6**A). We curated a comprehensive training dataset comprising compound structures and associated gene expression profiles from publicly available resources, including LINCS, DrugBank, and ChEMBL. Chemical structures were represented as SMILES strings, converted into grammar trees, and encoded into one‐hot arrays. These arrays were subjected to dimensionality reduction using a variational autoencoder (VAE) to generate input vectors (X). Differentially expressed genes (DEGs) associated with each compound were likewise encoded as one‐hot vectors to serve as output labels (Y). Feature selection focused on ten key *NF‐κB*‐associated inflammatory genes (*IL1B*, *IL6*, *TNF*, *PTGS2*, and *NOS2*), along with *Egr1*, *Ifnb1*, *ISG15*, *IFIT3*, and *CXCL10, which serve* as a representative marker of innate immune activation, yielding 10 target genes central to inflammation and antiviral defense. VDLIN consists of two major components. First, a three‐layer 1D CNN is used to extract structural features from compound embeddings. These are passed through a dense layer to generate mean and radius vectors, defining a high‐dimensional spherical latent space. A latent vector is then sampled and input into a four‐layer dense network, which predicts the expression levels of the 10 target genes.

To rigorously evaluate the performance advantages of VDLIN, we conducted a comprehensive benchmarking analysis against five widely used conventional machine learning algorithms: Support Vector Machine (SVM) with a radial basis function (RBF) kernel, k‐Nearest Neighbors (kNN), Logistic Regression, Random Forest (RF), and Gradient Boosting (GB). All models were trained using identical input features and consistent train‐test splits, with hyperparameters optimized via grid search and cross‐validation to ensure a fair comparison. As shown in Figure 6B, VDLIN achieved high predictive accuracy, with an area under the ROC curve (AUC) of 0.931 and a true positive rate (TPR) of 92.31%. Furthermore, as summarized in Figure 6C, VDLIN consistently outperformed all traditional models across key evaluation metrics. Specifically, it achieved an AUC 5.0% higher than the best‐performing baseline model (Gradient Boosting, AUC = 0.873), and an F1‐score of 0.925, representing a 3.2% improvement over Gradient Boosting (F1 = 0.893). This indicates a superior balance between precision and recall, particularly under class‐imbalanced conditions (Figure S11A, Supporting Information). To further assess model robustness, we introduced increasing levels of synthetic label noise (10%, 20%, and 30%) and evaluated the resulting performance degradation. VDLIN exhibited significantly greater resilience, with only a 4.3% reduction in F1‐score under 30% noise, compared to 9.8% for Gradient Boosting and 11.2% for SVM (Figure S11B, Supporting Information). This robustness is likely attributable to VDLIN's deep convolutional architecture, which effectively captures high‐level nonlinear dependencies while mitigating overfitting and noise sensitivity through regularization and dropout mechanisms. Collectively, these results underscore the superior predictive performance, robustness, and generalizability of VDLIN over a broad range of conventional machine learning models, highlighting its potential as a powerful tool for identifying immune‐related compounds that not only exhibit anti‐inflammatory properties similar to MCB but also possess the capacity to activate the type I interferon (IFN‐I) response robustly.

Next, we applied VDLIN to screen small molecules from the CAS compound database. Candidates with the highest predicted immunomodulatory potential were selected for experimental validation. Among these, one compound, designated Co7, exhibited robust dual activity: suppressing *NF‐κB*‐mediated inflammation while enhancing *Egr1*‐driven innate immune activation. The chemical structure of Co7 is shown in Figure S11C (Supporting Information). In vitro and in vivo assays confirmed VDLIN's predictions. Co7 robustly induced *Ifnb1* expression in RAW 264.7 and J774 macrophage cell lines, as well as in primary bone marrow‐derived macrophages (BMDMs) and peritoneal macrophages (PMs), with expression peaking at 3 h post‐treatment (Figure 6D). These findings validate VDLIN as a robust framework for rational drug discovery targeting innate immune regulation.

### 2.6 Co7 Enhances the Innate Immune Response Through Toll‐Like Receptor 4

**Figure 7:**
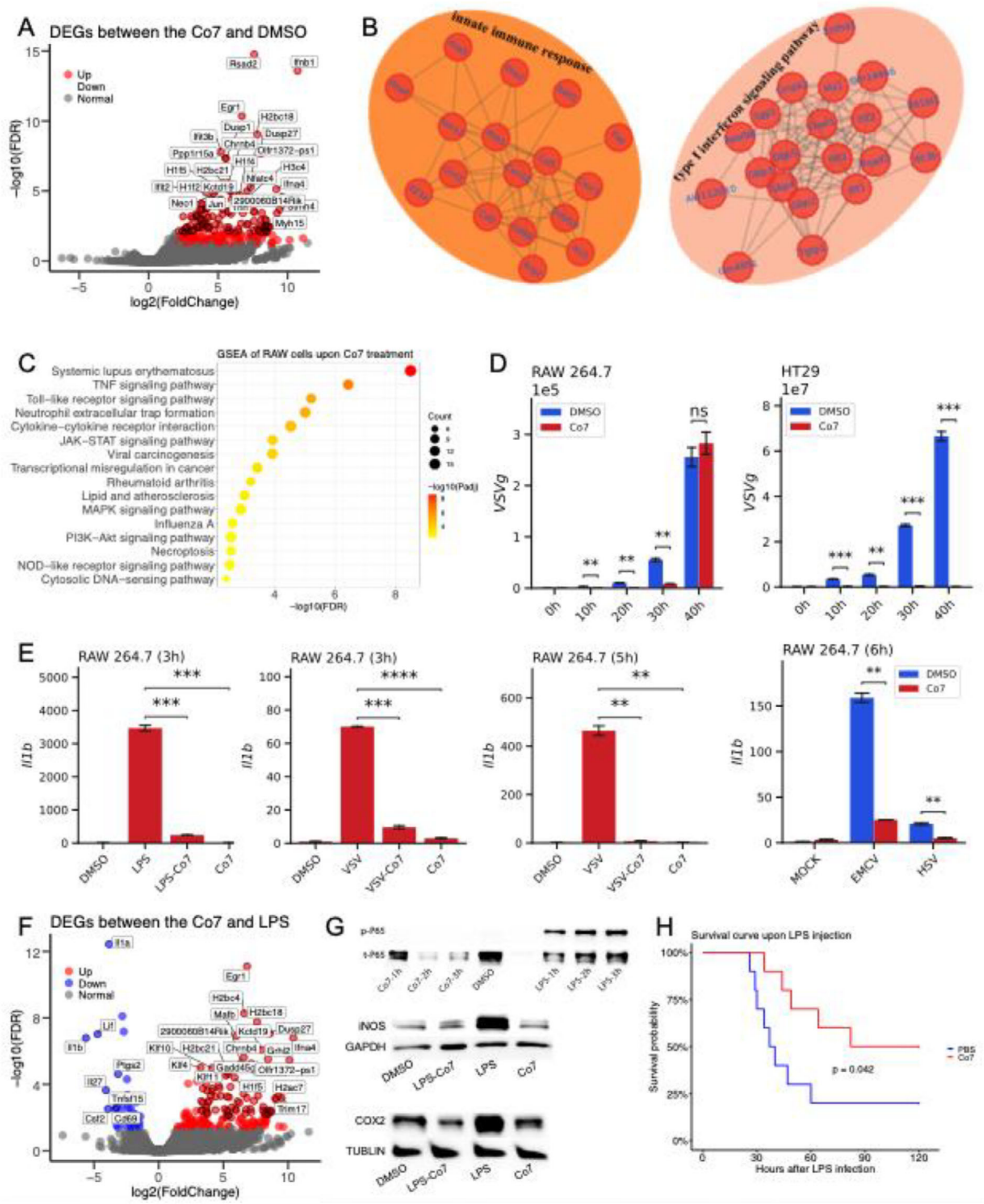
Co7 induces *Ifnb1* expression via the TLR4 signaling pathway. A) Volcano plot illustrating the distribution of differentially expressed genes (DEGs) between the Co7 and DMSO treatment groups after 3 h in RAW 264.7 cells. Fold changes are presented as log_2_ transformations. Red dots represent DEGs upregulated in the Co7 group. B) Protein‐protein interaction (PPI) network analysis of differentially expressed genes (DEGs) in the Co7‐treated group compared to the DMSO group, revealing significant enrichment in pathways associated with the innate immune response and type I interferon signaling. C) KEGG pathway enrichment analysis of DEGs induced by Co7, highlighting associations with innate immune response pathways. D) Co7 (50 µmol/L) exhibiting strong antiviral effects against VSV in RAW 264.7 and HT29 cells. E) Co7 significantly reduced the inflammatory response induced by LPS, VSV, EMCV, and HSV in RAW 264.7 cells. F) Volcano plot representing the differential gene expression analysis between Co7‐ and LPS‐treated RAW 264.7 cells after 3 h of treatment. Fold changes are displayed as log_2_ transformations. Red dots indicate genes upregulated in the Co7 group, while blue dots represent downregulated genes compared to LPS treatment. G) Western blot analysis demonstrating that Co7 inhibited the expression of iNOS and COX2, as well as the phosphorylation of NF‐κB‐P65 at the protein level in RAW 264.7 cells. H) Co7 significantly reduced the mortality rate in mice (n = 10 per group) following LPS challenge (20 mg/kg), compared to the PBS control group. RT‐qPCR data were presented as means ± SEM from three independent experiments. Statistical significance was determined using one‐way ANOVA with Bonferroni's multiple comparisons test (left three panels in E), paired‐samples t‐test (D, right panel of EMCV and HSV in E), or the log‐rank test (H). *P < 0.05, **P < 0.01, and ***P < 0.001.

To delineate the molecular target and signaling pathway through which Co7 triggers an *Egr1*‐dependent innate immune response, we performed bulk RNA sequencing on RAW264.7 cells treated with DMSO or Co7. The volcano plot (**Figure** **7**A) revealed significant upregulation of *Egr1*, *Ifnb1*, *Ifna4*, and ISGs including *Rsad2*, *Ifit2*, and *Ifit3b*, in the Co7 group. PPI network analysis (Figure 7B and Figure S11D, Supporting Information) showed that Co7‐induced DEGs were enriched in innate immune and type I interferon signaling pathways. GSEA further indicated activation of canonical pathways, including TNF signaling, Toll‐like receptor (TLR) signaling, MAPK signaling, NOD‐like receptor signaling, and cytosolic DNA sensing (Figure 7C).

We then applied pharmacological inhibitors and genetic knockouts to identify the upstream PRR mediating Co7 responses. Inhibitors of TLR4, TRIF, and TBK1/IKKε significantly suppressed *Ifnb1* induction in RAW264.7 and J774 cells (Figure S11E,F, Supporting Information). Consistently, BMDMs and peritoneal macrophages from *Tlr4* or *Trif* knockout mice exhibited markedly reduced *Ifnb1* expression in response to Co7 (Figure S11G,H, Supporting Information), confirming that Co7 activates the TLR4–TRIF–TBK1 axis. Given that MCB lacks antiviral properties, we next tested Co7's antiviral efficacy. Pretreatment with Co7 significantly inhibited vesicular stomatitis virus (VSV) infection in RAW264.7 and HT29 cells (MOI = 0.1), demonstrating potent antiviral activity (Figure 7D). These findings indicate that Co7 enhances type I interferon responses and ISG expression, contributing to antiviral defense. Since MCB suppresses inflammation via NF‐κB inhibition, we hypothesized that Co7 may retain or enhance this property. Indeed, Co7 significantly attenuated inflammatory responses induced by LPS, VSV, EMCV, and HSV (Figure 7E), suggesting broad anti‐inflammatory activity.

We performed RNA‐seq on LPS‐ and Co7‐treated RAW264.7 cells to further assess this effect. Compared to LPS alone, Co7 upregulated *Egr1*, *Ifnb1*, *Ifna4*, and several histone genes (*H2ac7*, *H1f5*, *H2bc4*, *H2bc11*, *H2bc18*) (Figure 7F). In contrast, classical pro‐inflammatory genes were downregulated, including *Il1a*, *Il1b*, *Il27*, *Csf3*, and *Ptgs2*. Western blotting further confirmed reduced expression of iNOS and COX2 following Co7 treatment (Figure 7G). We next assessed NF‐κB activity by measuring phosphorylation of p65. As expected, LPS induced robust p65 phosphorylation, whereas Co7 did not (Figure 7G), suggesting Co7 suppresses NF‐κB activation. Finally, in an LPS‐induced sepsis model (20 mg/kg), Co7 treatment significantly improved survival (Figure 7H), supporting its in vivo anti‐inflammatory efficacy. In summary, Co7, a derivative of MCB, exhibits dual immunomodulatory activities—suppressing NF‐κB‐driven inflammation while enhancing TLR4–TRIF–TBK1‐mediated type I interferon responses. These findings validate VDLIN as an effective strategy for identifying compounds with combined anti‐inflammatory and immunostimulatory potential in the treatment of immune‐related disorders.

## 3 Discussion

Clinical studies during the COVID‐19 pandemic indicated that methylcobalamin (MCB), an active form of Vitamin B12, may mitigate the cytokine storm associated with SARS‐CoV‐2 infection.^[^ ^24^, ^25^ ^]^ Subsequent research has revealed that MCB exerts anti‐inflammatory effects by methylating the promoter regions of inflammatory cytokines.^[^ ^25^, ^26^ ^]^ However, the specificity of this methylation and its impact on innate immune responses remain unclear. In this study, we validated MCB's anti‐inflammatory properties in cell and animal models treated with LPS. Notably, MCB also impaired macrophage‐mediated innate immune responses, potentially compromising antiviral defenses. To clarify the mechanisms underlying MCB's dual effects, we employed multi‐omics approaches, including RNA‐seq, ATAC‐seq, and CUT&Tag, to investigate the impact of MCB on transcriptome dynamics and chromatin accessibility following LPS induction. Our findings demonstrate that MCB reduces chromatin accessibility at NF‐κB and EGR1 binding sites, leading to decreased IFNB1 production and facilitating immune evasion. To address these limitations, we developed a deep learning model, VDLIN, which identified a novel compound, “Co7.”

Our RNA‐seq results support MCB's potent anti‐inflammatory role, demonstrating downregulation of inflammatory cytokines such as *Il1b*, *Il6*, and *Tnf*. This finding aligns with our ATAC‐seq analysis, which revealed that MCB significantly reduces LPS‐induced chromatin accessibility, particularly in regions associated with pro‐inflammatory transcription factors like NF‐κB. Notably, the accessibility and expression of key regulators, including *Egr1*, *Egr2*, and *Egr3*, were diminished after MCB treatment. The Early Growth Response (Egr) gene family (*Egr1*, *Egr2*, *Egr3*, and *Egr4*) encodes transcription factors activated by growth factors and neuronal activity, regulating essential processes such as proliferation, differentiation, and apoptosis.^[^ ^36^, ^37^ ^]^ Recent studies underscore *Egr1*’s role in regulating innate immunity and inflammation, highlighting its therapeutic potential.^[^ ^38^, ^39^, ^40^ ^]^ Our CUT&Tag analysis and functional experiments further validated *Egr1*’s regulatory roles in the innate immune response following MCB treatment. Additionally, ATAC‐seq footprinting analyses revealed significant enrichment of transcription factors, such as *CEBP‐1* and *ARID5A*, in the MCB‐treated group, which may function as molecular switches regulating immune responses, antiviral defenses, and suppressing excessive inflammation.^[^ ^41^, ^42^ ^]^ However, the mechanisms by which MCB decreases chromatin accessibility at these sites require further investigation.

Recent studies have demonstrated the transformative potential of machine learning (ML) in drug discovery, enabling predictions of biological activity and the identification of novel compounds.^[^ ^27^, ^28^, ^31^ ^]^ Supervised models, such as Random Forest and Support Vector Machines, are robust but face scalability and interpretability challenges.^[^ ^34^ ^]^ In contrast, deep learning models, including Convolutional Neural Networks (CNNs) and Recurrent Neural Networks (RNNs), excel in capturing complex molecular relationships but require extensive datasets and computational resources.^[^ ^43^ ^]^ In this study, we developed the CNN model VDLIN using TensorFlow and Keras, targeting ten marker genes downstream of NF‐κB and EGR1. VDLIN demonstrated excellent screening performance, validated by methods such as the confusion matrix and receiver operating characteristic (ROC) curve. Co7, identified through the CNN model VDLIN, is a potent modulator of immune signaling, particularly influencing type I interferon pathways. The inhibition of the TLR4 pathway significantly reduced Co7‐induced *Ifnb1* expression in various macrophage models, including RAW 264.7, J774, BMDMs, and PMs. This suggests that Co7's immunomodulatory effects depend heavily on TLR4‐TRIF signaling, positioning it as a potential therapeutic agent for regulating innate immunity. A key distinction between Co7 and MCB is their regulation of *Egr1* expression; while MCB inhibits LPS‐induced *Egr1*, Co7 significantly activates it, resulting in levels that exceed those induced by LPS alone. The mechanisms behind Co7's activation of *Egr1* and its contribution to Co7's anti‐inflammatory effects require further investigation. Additionally, more experiments are needed to assess Co7's efficacy in treating cold tumors resistant to PD‐1 checkpoint therapy,^[^ ^44^ ^]^ given its robust induction of *Ifnb1*. In conclusion, our findings demonstrate that Co7, through TLR4‐mediated modulation of transcriptional landscapes, exerts a dual effect on immune signaling—attenuating harmful pro‐inflammatory responses while enhancing antiviral defenses. This dual capacity offers a promising therapeutic avenue for managing hyper‐inflammatory conditions and improving antiviral immunity. Future research should explore the therapeutic effects and long‐term safety of Co7 in a broader range of viral infections and inflammatory disease models.

While the current study centers on MCB as a proof‐of‐concept compound, the architecture of the VDLIN model was intentionally designed to support broader applications beyond this specific context. In this work, VDLIN was trained using transcriptomic signatures associated with MCB‐induced immunomodulation, with the primary goal of identifying compounds that mimic its dual regulatory functions—namely, suppression of pro‐inflammatory cytokines and activation of type I interferon (IFN‐I) responses. This framework successfully prioritized the candidate compound Co7, which was experimentally validated to recapitulate key aspects of MCB's activity. Importantly, although VDLIN was tailored in this study to discover MCB‐related compounds, the model itself is inherently modular. By redefining the output gene set to reflect alternative biological programs—such as anti‐tumor immunity, metabolic reprogramming, or viral defense—the same deep learning architecture can be retrained to serve diverse compound screening objectives. This versatility is demonstrated by our development of a second model, VDLIN‐V2 (https://github.com/fynn‐guo/VDLIN_Model/blob/main/VDLIN‐V2.ipynb), which was specifically adapted to prioritize compounds with potential anti‐cancer activity. VDLIN‐V2 integrates tumor‐associated transcriptional signatures and, similar to the original VDLIN, uses the SMILES (Simplified Molecular Input Line Entry System) descriptor of each compound as its input. Although the detailed results of this application are beyond the scope of the present study, they highlight the platform's extensibility. Taken together, our findings demonstrate that VDLIN not only provides a robust and biologically informed strategy for identifying MCB‐related immunomodulatory compounds but also establishes a flexible framework for mechanism‐driven compound discovery across a range of immunological and pathological conditions.

As an essential cofactor for methionine synthase, MCB plays a pivotal role in one‐carbon metabolism, serving as a methyl donor for critical biological processes, including DNA and protein methylation. Its immunomodulatory effects are mediated indirectly through epigenetic regulation, particularly via methylation‐dependent modulation of inflammatory gene promoters. The cellular uptake of MCB is facilitated by the transcobalamin II receptor (TCII‐R), consistent with its physiological role as a vitamin B12 derivative. In contrast, Co7 represents a synthetically engineered cobalt complex with a distinct chemical architecture. Unlike MCB's corrin ring structure, Co7 incorporates a Phosphines‐Nitrogen‐Phosphines (PNP)‐pincer ligand system, reflecting its rational design as a targeted immunomodulator. This structural divergence underlies fundamentally different mechanisms of action: while MCB participates in metabolic pathways, Co7 directly modulates pattern recognition receptor signaling, particularly through TLR4 interactions. Our integrated transcriptomic and epigenomic analyses reveal contrasting immunoregulatory mechanisms between these cobalt‐containing compounds. MCB demonstrates broad suppressive effects on inflammatory and antiviral innate immune responses, achieved through transcriptional and chromatin‐level reprogramming. This global suppression aligns with its role in maintaining metabolic homeostasis through methylation‐dependent gene silencing. Conversely, Co7 exhibits a more nuanced, pathway‐specific immunomodulation. It displays dual regulatory capacity:^[^ ^1^ ^]^ suppression of pathological inflammatory responses while^[^ ^2^ ^]^ potentiation of antiviral defenses, notably through enhancement of type I interferon responses. This selective immunomodulation stems from its targeted interaction with innate immune signaling pathways, distinct from MCB's epigenetic mechanisms. The comparative analysis highlights that cobalt coordination chemistry alone does not determine biological activity. Instead, the distinct ligand environments—corrin ring in MCB versus PNP‐pincer in Co7—create fundamentally different molecular entities with divergent biological targets and effects. This structural differentiation explains why Co7's immunomodulatory profile extends beyond the scope of traditional cobalamin derivatives. These findings position Co7 as a prototype for a novel class of immunoregulatory agents, distinguished from vitamin B12 derivatives by its synthetic architecture and targeted mechanism of action. The study underscores the importance of molecular context in metal‐containing compounds, where identical central atoms can yield dramatically different biological outcomes depending on their coordination environment.

## 4 Experimental Section

### Reagents and Antibodies

Rabbit anti‐COX2 antibodies were purchased from Wanleibio. Rabbit antibodies against iNOS (catalog no. 80517‐1‐RR) and Beta Tubulin (catalog no. 10094‐1‐AP), along with mouse antibodies targeting Beta Actin (catalog no. 66009‐1‐Ig), GAPDH (catalog no. 60004‐1‐Ig), and the DYKDDDDK tag (catalog no. 66008‐4‐Ig), were procured from Proteintech. Additional antibodies, including rabbit phospho‐NF‐κB p65 (Ser536) (93H1) (catalog no. 3033) and EGR1 (15F7) (catalog no. 4153), were obtained from Cell Signaling Technology. Secondary detection utilized HRP‐conjugated Affinipure Goat Anti‐Rabbit IgG (H+L) (catalog no. SA00001‐2) and HRP‐conjugated Affinipure Goat Anti‐Mouse IgG (H+L) (catalog no. SA00001‐1), also from Proteintech. ABclonal supplied active recombinant mouse CSF‐2/GM‐CSF protein (catalog no. RP01206), while Lipopolysaccharides (LPS) (catalog no. HY‐D1056) and Methylcobalamin (catalog no. HY‐B0586) were sourced from MedChemExpress. Dimethyl sulfoxide (DMSO) (catalog no. D8371) was purchased from Solarbio.

### Cell Culture and Viral Infection

The RAW264.7, J774A.1, and HEK293T cell lines were sourced from the American Type Culture Collection (ATCC). Bone marrow‐derived macrophages (BMDMs) were isolated from the femurs and tibiae of 8‐week‐old mice and cultured with granulocyte‐macrophage colony‐stimulating factor (GM‐CSF, RP01206, ABclonal) for 7 days to induce macrophage differentiation. Peritoneal macrophages (PMs) were collected from 8‐week‐old mice following intraperitoneal injection of 2 ml thioglycolate broth solution, facilitating macrophage recruitment. Three days post‐injection, 5 mL of phosphate‐buffered saline (PBS) was injected into the peritoneal cavity, followed by gentle abdominal massage to promote macrophage detachment. HEK293T, RAW264.7, J774A.1, and BMDMs were cultured in Dulbecco's Modified Eagle's Medium (DMEM) (EallBio, 03.1002C), while PMs were maintained in RPMI 1640 (EallBio, 03.4001C). All culture media were supplemented with 10% fetal bovine serum (FBS) and 100 U/ml penicillin‐streptomycin for optimal cell growth and maintenance.

Subsequently, the cells reaching 70–80% confluence were infected with vesicular stomatitis virus (VSV) at a multiplicity of infection (MOI) of 0.1, herpes simplex virus‐1 (HSV‐1, F strain, MOI of 0.5), encephalomyocarditis virus (EMCV, MOI of 0.1), or treated with lipopolysaccharide (LPS, 1 µg/mL) for various durations. After viral infection for 1 h, the media were removed, and fresh medium was added to the cultures for further analysis.^[^ ^45^ ^]^

### Mice and Construction of a Sepsis Model via LPS Injection

All animal care and procedures adhered to the Guide for the Care and Use of Laboratory Animals established by the Chinese Association for Laboratory Animal Science. The Animal Care Committee of Peking University Health Science Center approved all handling procedures (permit number LA 2 016 240). TLR4^−/−^ and TRIF^−/−^ mice on a C57BL/6J background were obtained from the Institute of Experimental Animals, Chinese Academy of Medical Sciences. In contrast, wild‐type (WT) C57BL/6J mice were purchased from the Department of Laboratory Animal Science at Peking University Health Science Center. All animals had access to clean water and nutritious feed. The primers utilized for genotyping included TLR4 KO (forward: 5′‐TGCTCACACCATCATCAC‐3′; reverse: 5′‐CATGTACTAGGTTCGTCAGA‐3′) and TRIF KO (forward: 5′‐CTCCAGTCTCTTCCCCACAG‐3′; reverse: 5′‐GTCTCAAGCTGGGTCCAACT‐3′).

This study employed lipopolysaccharide (LPS)‐induced sepsis models to investigate systemic inflammatory responses characteristic of sepsis. A solution of LPS was prepared in sterile saline at a concentration of 20 mg/kg body weight for the WT C57BL/6J mice (n = 20) and administered intraperitoneally using sterile insulin syringes. After LPS injection, a subset of mice (n = 10) received an intraperitoneal injection of either methylcobalamin (MCB) (30 mg/kg) or Co7 (10 mg/kg). At the same time, the control group (n = 10) was injected with an equivalent volume of PBS. Survival times were recorded post‐injection, and blood, spleen, and lung tissues were subsequently harvested for further analysis.

### RNA Extraction, Reverse Transcription, and Real‐Time Quantitative PCR

RNA extraction from cells or tissues following various treatments or infections was performed using TRIzol reagent (TIANGEN, A0123A01). The purified RNA was then reverse‐transcribed into cDNA using HiScript II RT SuperMix (Vazyme, R223‐01). Target gene expression was quantified using SYBR Green qMix (Vazyme, Q311) in a quantitative reverse transcription PCR (RT‐qPCR) assay. The relative expression levels of target mRNAs were normalized to the housekeeping gene *Actb*. Detailed primer sequences used in this study are provided in Table S1 (Supporting Information).

### Total Protein Extraction and Western Blot Analysis

Cells were lysed using RIPA Lysis Buffer (Strong) (MedChemExpress, HY‐K1001), which was supplemented with protease inhibitors, including an EDTA‐Free Protease Inhibitor Cocktail, Phosphatase Inhibitor Cocktail I (100× in DMSO), and Phosphatase Inhibitor Cocktail III (100× in DMSO). Clarified cell extracts (10 to 30 micrograms) were resolved on SDS‐polyacrylamide gels and then transferred onto nitrocellulose membranes (Beyotime, FFN08). After blocking, the membranes were incubated with specific primary antibodies. The bound secondary antibodies were detected using the enhanced chemiluminescence (ECL) method (EallBio, 07.10009‐50).

### Pharmacological Inhibition of Proteins

Cells were treated with specific inhibitors upon reaching 70–80% confluence to investigate the role of various signaling pathways. Each inhibitor was dissolved in anhydrous DMSO and diluted to its respective working concentration: C29 (10 µM, S6597, Selleck) served as a TLR2 inhibitor; Procyanidin B1 (30 µM, HY‐N0795, MedChemExpress) acted as a TLR4 inhibitor; MyD88‐IN‐1 (30 µM, HY‐149992, MedChemExpress) was used to inhibit MyD88; Pepinh‐TRIF TFA (30 µM, HY‐P2565, MedChemExpress) functioned as a TRIF inhibitor; and GSK8612 (5 µM, T5540, TargetMol) inhibited TBK1/IKKε. Following treatment, cells were incubated at 37 °C for 3 h. Control groups were treated with DMSO at a concentration of less than 0.1%. This systematic approach assessed specific pathway contributions to the observed cellular responses.

### Construction of Egr1 Expression Plasmid

Mouse *Egr1* cDNA was cloned into the pCDNA3.1_3XFlag expression vector using a seamless cloning and assembly kit (Transgen, CU101‐01), following the manufacturer's standard protocol. The coding sequences were verified in their entirety through Sanger sequencing to ensure the accuracy and integrity of the cloned plasmid.

### Short Hairpin RNA (shRNA) of Egr1 for Gene Silencing

Short hairpin RNA (shRNA) technology is widely employed to silence specific target genes in cells. In this study, the pLKO.1 plasmid as a vector, which contains EcoRI and AgeI restriction enzyme cutting sites, was utilized. The shRNA sequences designed to knock down *Egr1* were obtained from the Sigma–Aldrich online database (https://www.sigmaaldrich.cn/CN/zh/product/sigma/shrna) and are detailed in Table S2 (Supporting Information). Initially, three pairs of shRNA were designed for *Egr1*; however, after validation, the second pair was selected for further experiments due to its significantly higher knockdown efficiency. The sequences for *Egr1*‐shRNA‐pair2 were *CCGGCACTCCACTATCCACTATTAACTCGAGTTAATAGTGGATAGTGGAGTGTTTTTG* and *AATTCAAAAACACTCCACTATCCACTATTAACTCGAGTTAATAGTGGATAGTGGAGTG*. The non‐targeting shRNA (negative control shRNA, also named “scramble shRNA”) from Sigma–Aldrich was utilized as a control. Transfections were performed at a final concentration of 10 nM using Lipofectamine RNAiMAX Transfection Reagent (catalog number 13 778 030; Thermo Fisher Scientific), following the manufacturer's instructions. After transfections, the cells were split and selected with 5 mg/ml puromycin for two weeks. Following selection, stable expressing clones were isolated by limiting dilution and subsequently expanded for further analysis.

### RNA‐seq and Data Analysis

Total RNA was extracted using the high‐throughput RNA extraction kit (TIANGEN, A0123A01). Quality control, library preparation, and sequencing were performed by Suzhou GENEWIZ Biotechnology Company (https://www.genewiz.com.cn/), following established standard protocols. Data analysis was carried out in‐house. Sequencing raw data in FASTQ format underwent quality control using FastQC (v0.11.9) and Trim‐Galore (v0.6.4) software to generate clean data for downstream analysis. Trim‐Galore was executed with the following parameters: trim_galore –gzip –trim‐n –phred33 ‐j 7 –paired ${var}_1.fq.gz ${var}_2.fq.gz ‐o $wrk_dir/clean_result/. Clean reads were aligned to the mm10 reference genome using Subread (v2.0.0) with the following parameters: subread‐align ‐i $idx_dir ‐r $cle_dir/${var}_1_val_1.fq.gz ‐R $cle_dir/${var}_2_val_2.fq.gz ‐o $aln_dir/${var}.bam ‐T 30 ‐t 0.^[^ ^46^ ^]^ The gene count matrix was generated using featureCounts (v2.0.0) with the following parameters: featureCounts ‐p ‐t exon ‐g gene_id ‐a $gtf_dir ‐o $cnt_dir/count_refGene $aln_dir/*.bam ‐T 29.^[^ ^47^ ^]^ The count data were normalized using the Fragments Per Kilobase Million (FPKM) formula. Differential expression analysis was conducted using the DESeq2 R package (v1.38.3),^[^ ^48^ ^]^ with the thresholds set at |log2(FoldChange)| > 2 and *p*‐value < 0.05 to identify significantly differentially expressed genes (DEGs).

### GO Annotation and KEGG Pathway Enrichment Analysis

This study subjected DEGs to enrichment analysis through Gene Ontology (GO) annotation and Kyoto Encyclopedia of Genes and Genomes (KEGG) pathway analysis. This analysis was performed using the clusterProfiler R package,^[^ ^49^ ^]^ explicitly employing the enrichGO and enrichKEGG functions. Alternatively, the online database DAVID (https://david.ncifcrf.gov/summary.jsp) (V6.8) was utilized for the same purpose.^[^ ^50^ ^]^ Significance thresholds were established, with GO and KEGG terms having false discovery rates (FDR) less than 0.01 considered indicative of meaningful enrichments.

### Gene Set Enrichment Analysis (GSEA)

Gene Set Enrichment Analysis (GSEA) is a computational approach used to determine whether predefined gene sets exhibit statistically significant and coordinated differences between two biological states.^[^ ^51^ ^]^ Unlike traditional methods that focus solely on differentially expressed genes (DEGs), GSEA incorporates all genes in the analysis, regardless of their significance levels. In this study, GSEA was conducted using the clusterProfiler package, and the function gseaplot2 from the same package was employed to visualize the enrichment results.

### Protein–Protein Interaction (PPI) Network Analysis

Protein–protein interaction (PPI) network analysis is a computational approach to investigate and map the interactions between proteins within a biological system.^[^ ^52^ ^]^ For this analysis, the online database STRING (https://string‐db.org/) with default parameters was utilized. The input consisted of differentially expressed genes (DEGs), and the output was a network representing the interactions between the proteins encoded by these DEGs. The resulting PPI network was visualized using Cytoscape software (v3.10.2).^[^ ^53^ ^]^

### Transcription Factor Enrichment Analysis (TFEA)

Transcription factor enrichment analysis (TFEA) is a computational method used to identify transcription factors (TFs) that may regulate differentially expressed genes (DEGs). For this analysis, iRegulon (http://iregulon.aertslab.org/tutorial.html), a Cytoscape plug‐in specifically designed for TFEA was utilized. The input for iRegulon is a list of DEGs, and the output consists of a ranked list of predicted TFs based on enrichment scores.^[^ ^54^ ^]^ The results of TFEA can be directly visualized and further explored using Cytoscape's integrated visualization tools (v3.10.2).

### ATAC‐seq

A high‐throughput sequencing methodology based on transposase‐mediated chromatin accessibility profiling (ATAC‐seq) was employed.^[^ ^55^ ^]^ This technique utilizes the specificity of Tn5 transposase to selectively cleave accessible regions of chromatin. The transposase, preloaded with DNA sequence adapters, was incubated with isolated cell nuclei, enabling the targeted insertion of adapters into open chromatin regions. Subsequently, indexed primers were utilized for PCR amplification to construct sequencing libraries. The resultant libraries, following sequencing, provided comprehensive insights into DNA regions associated with chromatin accessibility. This experiment was performed using the Hyperactive ATAC‐Seq Library Prep Kit (Vazyme Biotech, TD711), with sequencing services conducted by GENEWIZ Biotechnology company. (Suzhou, China).

### CUT&Tag

In this study, cells were immobilized using Concanavalin A‐coated Magnetic Beads Pro (ConA Beads Pro) and permeabilized with the nonionic detergent digitonin. The target protein was then detected using a specific primary antibody against EGR1, followed by a corresponding secondary antibody and Protein A/G, which formed an immunocomplex. To tether the Tn5 transposase to the target protein, it was fused to Protein A/G and guided by the antibody complex. Upon activation with Mg^2^⁺, the Tn5 transposase catalyzed targeted DNA cleavage, simultaneously inserting adapter sequences at both ends of the fragmented DNA. These DNA fragments were then amplified via PCR for library construction. The entire process was performed using the CUT&Tag Library Prep Kit (Transgen, KP172), with sequencing services provided by GENEWIZ Biotechnology company (Suzhou, China).

### Data Analysis of ATAC‐seq and CUT&Tag

For ATAC‐seq and CUT&Tag high‐throughput data, sequenced by GENEWIZ Biotechnology, raw fastq data underwent quality control using Trim‐Galore (v0.6.4). Filtered reads were then aligned and quantified against the mouse genome mm10 using the bowtie2 aligner (v2.3.5.1),^[^ ^56^ ^]^ generating SAM or BAM files. The bowtie2 alignment parameters were: bowtie2 –very‐sensitive ‐X 2000 ‐x $Bowtie2Index ‐1 $cln_res/${sample}_1_val_1.fq.gz ‐2 $cln_res/${sample}_2_val_2.fq.gz ‐p $PPN | samtools view ‐buSh ‐@ $PPN | samtools sort ‐@ $PPN ‐O BAM ‐o $aln_res/${sample}.sorted.bam. Sorting and indexing of SAM/BAM files were completed using samtools (v1.10) with the command: samtools index ‐@ $PPN $aln_res/${sample}.sorted.bam.^[^ ^57^ ^]^

Peak calling was performed using MACS3 (v3.0.0a5) with default parameters, yielding peak files in BED format for each sample.^[^ ^58^ ^]^ For downstream analysis, different strategies were applied to ATAC‐seq and CUT&Tag data. ATAC‐seq peaks were merged across all samples using the bedtools merge (v2.31.1) function,^[^ ^59^ ^]^ followed by normalization with bamCoverage (v3.3.2) in the deepTools suite^[^ ^60^ ^]^ using the command: bamCoverage –bam $var ‐o ${var%.*}.bw –binSize 100 –normalizeUsing RPKM –effectiveGenomeSize 2 864 785 220 –ignoreForNormalization chrM –extendReads. For CUT&Tag data, reads in peak regions were normalized by RPKM using the same bamCoverage parameters. Comparisons between treatment and input samples were done using bamCompare with the following command: bamCompare ‐b1 idx‐MUTMSCLACH.bam ‐b2 idx‐MUTMSCLAIN.bam ‐o bwResIpInput/MUTIPratioInput –binSize 145 –normalizeUsing RPKM –effectiveGenomeSize 2 864 785 220 –ignoreForNormalization chrM –extendReads ‐p 22 –scaleFactorsMethod None. Differential analysis for both ATAC‐seq and CUT&Tag was conducted using csaw R package (v1.38.0), while peak annotation was performed using the ChIP seeker R package (v1.34.1).^[^ ^61^ ^]^ Finally, peak visualization was achieved using Integrative Genomics Viewer (IGV) (v2.17.4).^[^ ^62^ ^]^

### GREAT Analysis

GREAT (genomic regions enrichment of annotations tool) is a powerful resource for functional enrichment analysis of unannotated genomic regions, especially in non‐coding areas. By associating these regions with nearby coding genes, GREAT allows researchers to infer potential biological functions.^[^ ^63^, ^64^ ^]^ This approach is beneficial for studying cis‐regulatory elements, such as the differentially accessible regions (DARs) identified through techniques like ATAC‐seq, ChIP‐seq, or CUT&Tag. By leveraging GREAT, insights into the functional roles of these unannotated regions could be gained, enhancing the understanding of genome regulation and gene expression control.

### Preprocessing and Encoding of Drugs‐Gene Expression Profiles Data

This study utilized a comprehensive dataset of gene expression profiles for small molecules sourced from the Library of Integrated Network‐Based Cellular Signatures (LINCS) project (https://lincsportal.ccs.miami.edu/signatures/datasets/), DrugBank (https://go.drugbank.com/), and ChEMBL (https://www.ebi.ac.uk/chembl/), encompassing both chemical structure and bioactivity data. Stringent data cleaning criteria were applied: molecules with fewer than seven replicates were excluded, and molecular SMILES were parsed using RDKit (v2024.03.5). Profiles for each molecule were averaged, disregarding plate, dose, treatment time, and cell line variations. The study focused on landmark genes related to inflammation and *Egr1*, induced by lipopolysaccharide (LPS) and methylcobalamin (MCB), yielding a final dataset of 7861 valid molecules, which was divided into training (5502) and test (2359) sets. Chemical structures of small molecules were represented as SMILES descriptors, which were transformed into grammar trees and subsequently converted into one‐hot encoded arrays. These molecular features were processed into fixed‐length vectors for input into a convolutional neural network (CNN). Dimensionality reduction was performed using a variational autoencoder (VAE), generating the input matrix (X). Concurrently, each molecule's effect on gene expression was encoded into one‐hot arrays, with “1” indicating gene upregulation, 0 indicating no significant change, and ‘‐1′ indicating downregulation, forming the output matrix (Y).

### Construction of VDLIN Model

The CNN‐based VDLIN model (Vitamin B12‐derived Deep Learning for Innate Immunity) was developed to predict the dual capacity of small molecules to suppress inflammatory responses while enhancing innate immune activation. Informed by the transcriptomic and molecular characteristics of Vitamin B12, the model was implemented using TensorFlow and Keras, and trained to infer compound‐induced gene expression profiles with high predictive accuracy. The model architecture was structured to capture molecular feature interactions and their corresponding gene expression patterns. Each small molecule was represented as a feature vector of dimensions (4717, 6042, 1). The model began with a Conv1D layer comprising 32 filters with a kernel size of 3, applying the ReLU activation function to extract relevant molecular features. This was followed by a MaxPooling1D layer to reduce dimensionality while preserving critical information. A second Conv1D layer with 64 filters, followed by another MaxPooling1D layer, further refined the feature extraction. The output from these convolutional layers was flattened into a one‐dimensional vector and passed through a Dense layer containing 128 neurons with ReLU activation. To prevent overfitting, a dropout rate of 0.5 was employed. Using a sigmoid activation function, a final Dense output layer predicted the expression status (upregulated or downregulated) of landmark genes. The model was compiled using the Adam optimizer with binary cross‐entropy loss for binary classification.

### Design Principles of the Scoring Algorithm

- Positive Regulation Indicators: For key genes in the IFN‐I signaling pathway (e.g., *Egr1*, *Ifnb1*, *ISG15*, *IFIT3*, *CXCL10*), if a gene is upregulated (ΔExpression > 0), it is awarded +10 points per gene.
- Negative Regulation Indicators: For major pro‐inflammatory cytokines (e.g., *IL1B*, *IL6*, *TNF*, *PTGS2*, *NOS2*), if a gene is downregulated (ΔExpression < 0), it is also awarded +10 points per gene.
- Theoretical Score Range: The total score ranges from 0 (no desired regulatory effects) to 100 (ideal regulation of all 10 target genes).
- Mathematical Expression:
(1) $$\mathrm{Score}=\sum_{i=1}^{5}\mathrm{II}\left(\Delta G_{i}^{IFN}>0\right)\times 10+\sum_{j=1}^{5}\mathrm{II}\left(\Delta G_{j}^{Inflame}<0\right)\times 10$$

where II is the indicator function, and ΔG represents the predicted gene expression change.

### Model Integration Strategy

This scoring function was incorporated into a multi‐task convolutional neural network (CNN) architecture with two main branches:

- Expression Regression Branch: Predicts the expression changes (Δ values) of the ten target genes.
- Scoring Branch: Computes the total immunomodulatory score based on the predicted expression changes using the scoring formula above.

During training, both objectives were optimized jointly. The total loss function is defined as:

(2) $$L=\alpha \cdot \mathrm{MSE}+\left(1-\alpha \right)\cdot \left[1-\sigma \left(\mathrm{Score}/100\right)\right]$$

where σ(⋅) denotes the sigmoid function, and α was set to 0.7 to balance the accuracy of expression prediction and score optimization.

### Screening Pipeline

(3) $$\sum_{i=1}^{5}\mathrm{II}\left(\Delta G_{i}^{IFN}>0\right)\geq 3,\sum_{j=i}^{5}\mathrm{II}\left(\Delta G_{j}^{Inflame}<0\right)\geq 3$$

- Primary Screening: Compounds with total scores ≥ 70 (significantly higher than the random background, >3.5 standard deviations above the mean) were selected as potential candidates.
- Secondary Screening: Compounds were required to meet the following robustness criteria for consistent gene‐level effects:

Only compounds that passed both criteria were ranked based on their total scores. Subsequently, the collaborators at the Department of Chemistry, Tsinghua University, synthesized high‐ranking compounds. Their activities were experimentally validated in cellular and animal models to ensure biological relevance and therapeutic potential.

### Hyperparameter Optimization

To ensure a rigorous and fair comparison between the proposed VDLIN model and conventional machine learning methods, a unified Bayesian hyperparameter optimization framework was employed based on Gaussian processes, implemented using the Scikit‐Optimize library. This strategy was selected for its superior sample efficiency, capacity to handle mixed‐type search spaces, and robustness in high‐dimensional optimization tasks. The optimization protocol was designed around three core principles: methodological consistency across models, exhaustive exploration of relevant hyperparameter spaces, and computational scalability.

Each model underwent a maximum of 100 optimization iterations, with early stopping triggered if no improvement in validation loss was observed for 20 consecutive steps. The objective function was the area under the receiver operating characteristic curve (AUC‐ROC), evaluated using threefold cross‐validation to reduce variance. A stratified 20% subset of the training data was consistently used as the validation set across all models. To enhance efficiency, all optimization tasks were parallelized across 16 CPU cores using the Joblib library. For the VDLIN model, a parameter space encompassing architectural and training‐related hyperparameters was explored. These included hidden layer dimensions ({64, 128, 256}), number of attention heads ({2, 4, 8}), learning rate (log‐uniform between 1 × 10^−4^ and 0.1), batch size ({32, 64, 128}), and dropout rate (uniform between 0.1 and 0.5).

Baseline models were optimized under identical protocols. For tree‐based methods, Random Forest was tuned over n_estimators (50–200), max_depth,^[^ ^3^, ^4^, ^5^, ^6^, ^7^, ^8^, ^9^, ^10^, ^11^, ^12^, ^13^, ^14^, ^15^ ^]^ and min_samples_split^[^ ^2^, ^3^, ^4^, ^5^, ^6^, ^7^, ^8^, ^9^, ^10^ ^]^; XGBoost was optimized for learning_rate (0.01–0.3), max_depth,^[^ ^3^, ^4^, ^5^, ^6^, ^7^, ^8^, ^9^, ^10^ ^]^ and subsample ratio (0.6–1.0). For linear models, logistic regression parameters included C (0.01–10, log‐uniform) and penalty type (“l1,” “l2”). Instance‐based models such as k‐nearest neighbors (k‐NN) were tuned over n_neighbors^[^ ^3^, ^4^, ^5^, ^6^, ^7^, ^8^, ^9^, ^10^, ^11^, ^12^, ^13^, ^14^, ^15^ ^]^ and distance metric (“euclidean,” “manhattan”). Kernel‐based support vector machines (SVMs) were explored across penalty parameters C (0.1–10), kernel coefficient γ (0.01–1), and kernel types (“linear,” “rbf”). This rigorous and standardized hyperparameter tuning procedure ensured that all model evaluations accurately reflected their true representational capacity, rather than being artifacts of suboptimal configuration.

### Analysis of the Performance of VDLIN

The performance of VDLIN was evaluated using several key metrics, including the confusion matrix, precision‐recall (PR) curve, receiver operating characteristic (ROC) curve, and the area under the ROC curve (AUC). These metrics provided a comprehensive assessment of the model's ability to predict gene expression profiles induced by small molecules, allowing for an in‐depth understanding of both its precision and recall, as well as its overall classification accuracy and discriminatory power. The confusion matrix offered insights into true positive, true negative, false positive, and false negative predictions, while the PR and ROC curves, along with the AUC, illustrated the model's trade‐offs between sensitivity and specificity across various thresholds. To evaluate the predictive performance and robustness of VDLIN in comparison with conventional machine learning algorithms, five baseline models were implemented: Support Vector Machine (SVM) with a radial basis function (RBF) kernel, k‐Nearest Neighbors (kNN), Logistic Regression, Random Forest (RF), and Gradient Boosting (GB). All models were trained using the same input features and identical training/testing splits. Hyperparameters for each model were optimized via grid search with five‐fold cross‐validation to ensure fair and reproducible comparisons. To assess model robustness, synthetic label noise was introduced into the training data at varying levels (10%, 20%, and 30%). For each noise level, class labels of a randomly selected proportion of training samples were permuted while keeping the test set unchanged. Models were retrained under each noise condition, and the resulting performance degradation was measured. Figure S9B (Supporting Information) presents the change in F1‐score across noise levels, highlighting the stability of VDLIN relative to baseline models.

### Statistical Analysis

In the present study, the Python package SciPy (https://pypi.org/project/scipy/) was used to conduct all the statistical analyses as indicated. Results are presented as the mean ± SEM. The *p* values < 0.05 were considered statistically significant (*), *p* values < 0.01, and *p* values < 0.001 were regarded as highly statistically significant (** and ***).

### Data Availability

The RNA‐seq, ATAC‐seq, and CUT&Tag datasets generated in this study have been deposited in the Gene Expression Omnibus (GEO) under the accession numbers GSE274237, GSE274235, and GSE274236, respectively. The source code used for model development and data analysis is publicly available at: https://github.com/fynn‐guo/VDLIN_Model.

## Conflict of Interest

The authors declare no conflict of interest.

## Supporting information



Supporting Information

Supplemental Table 1

Supplemental Table 2

## Data Availability

The data that support the findings of this study are available on request from the corresponding author. The data are not publicly available due to privacy or ethical restrictions.
