# Supplementary material for: VDLIN: A Deep Learning‐Based Platform for Methylcobalamin‐Inspired Immunomodulatory Compound Screening
Source: Adv Sci (Weinh). 2025 Oct 27;13(7):e13775. doi: 10.1002/advs.202413775 (PMC12866821; doi:10.1002/advs.202413775)
Supplement: Supplementary file 1 — Supporting Information [file ADVS-13-e13775-s003.docx]

Supplementary Figure 1. Methylcobalamin (MCB) suppresses LPS-induced cytokine and chemokine expression in RAW 264.7 cells. A) MCB treatment reduced LPS-induced *Il6* and *Ifnb1* expression in a concentration-dependent manner, with suppression observed at 2 μmol/L, 5 μmol/L, 10 μmol/L, 20 μmol/L, and 50 μmol/L. B) MCB inhibited *Il6* and *Nos2* expression in response to LPS stimulation across multiple time points, including 0 h, 1 h, 3 h, 6 h, 12 h, and 24 h. C) MCB significantly downregulated the expression of the chemokines *Ccl3* and *Ccl4*, as well as the pro-inflammatory cytokines *Lta* and *Ltb*, induced by LPS, as determined by RT-qPCR. D) MCB markedly suppressed the expression of interferon-stimulated genes (ISGs), including *Cxcl10*, *Cxcl11*, *Ifit1*, and *Isg15*, in response to LPS treatment, as determined by RT-qPCR. Data were obtained from three independent experiments and analyzed using RT-qPCR. Error bars represent the mean ± SEM. One-way ANOVA with Bonferroni's multiple comparisons test (A, C-D) or paired-samples T-test (B) was used to assess statistical significance. * *P* < 0.05, ** *P* < 0.01, and *** *P* < 0.001.

Supplementary Figure 2. Transcriptomic alterations induced by LPS and MCB treatment. A) Principal Component Analysis (PCA) demonstrating that MCB markedly reprogrammed the transcriptomic landscape in response to LPS stimulation. B) Volcano plot illustrating the distribution of differentially expressed genes (DEGs) between the LPS-MCB and LPS groups after 12-hour treatment in RAW 264.7 cells. The fold change values were log₂-transformed. Red dots indicate DEGs upregulated in the LPS-MCB group, whereas blue dots represent DEGs downregulated following MCB treatment. C) Gene Ontology (GO) enrichment analysis of upregulated differentially expressed genes (DEGs) in the LPS-MCB group compared to the LPS group following 12-hour treatment in RAW 264.7 cells. D) Kyoto Encyclopedia of Genes and Genomes (KEGG) pathway enrichment analysis of downregulated differentially expressed genes (DEGs) in the LPS-MCB group compared to the LPS group following 12-hour treatment. E) Gene Set Enrichment Analysis (GSEA) illustrating key transcriptomic pathways regulated by MCB in RAW 264.7 cells following 12-hour LPS stimulation.

Supplementary Figure 3. Enrichment analysis of differentially expressed genes (DEGs) between the LPS-MCB and LPS treatment groups after 12 hours in RAW 264.7 cells. A) Gene Ontology (GO) enrichment analysis of downregulated DEGs in the LPS-MCB group compared to the LPS group, revealing enrichment in pathways related to the inflammatory response, immune response, and cellular response to interleukin-1. B-C) Gene Set Enrichment Analysis (GSEA) showing key transcriptomic pathways downregulated by MCB in the context of LPS treatment, specifically *E2F targets* and the *G2M checkpoint*. D-E) GSEA highlighting key pathways upregulated by MCB in the context of LPS treatment, including *heme* and *bile acid metabolism*.

Supplementary Figure 4. Protein-protein interaction (PPI) network analysis of differentially expressed genes (DEGs) in the LPS-MCB group compared to the LPS group after 12-hour treatment in RAW 264.7 cells. A-B) PPI network analysis of downregulated DEGs in the LPS-MCB group, indicating significant enrichment in DNA replication and cell cycle pathways, with hub genes including the *Mcm* and *Kif* families. C) PPI network analysis of upregulated DEGs in the LPS-MCB group, demonstrating significant enrichment in lipid metabolic processes, containing hub genes such as *Adgre1*, *Itga7*, *Sdc3*, *Apoe*, *Lpin1*, and *Itgam*. D) Bar plot displaying the FPKM expression levels of selected inflammation-related genes downregulated by MCB, including *Ikbke*, *Pdgfb*, *Nod2*, and *Nlrp3*. E) Bar plot displaying the FPKM expression levels of selected autophagy-related genes upregulated by MCB treatment in RAW 264.7 cells, such as *Atg12*, *Atg14*, *Nbr1*, *Map1lc3a*, *Pink1*, and *Ulk1*. Statistical significance was determined by one-way ANOVA with Bonferroni’s multiple comparisons test (D-E). * *P* < 0.05, ** *P* < 0.01, and *** *P* < 0.001.

Supplementary Figure 5. Transcription factor enrichment analysis (TFEA) of differentially expressed genes (DEGs) between the LPS-MCB and LPS groups after 12 hours of treatment in RAW 264.7 cells. A) TFEA of upregulated DEGs in the LPS-MCB group relative to the LPS group identifies *Foxn3* as a potential transcription factor. Yellow nodes represent enriched transcription factors, while red nodes indicate upregulated DEGs specific to the LPS-MCB group. B-E) TFEA of downregulated DEGs in the LPS-MCB group relative to the LPS group identifies *Sfpi1*, *E2f1*, *Nfya*, and *Stat1* as potential transcription factors. Yellow nodes correspond to enriched transcription factors, while blue nodes represent downregulated DEGs in the LPS-MCB group.

Supplementary Figure 6. Statistical analysis of peaks identified by ATAC-seq among the LPS, LPS-MCB, and MCB groups compared to the DMSO group in RAW 264.7 cells upon 12-hour treatment. A-B) The number and ratio of peaks identified in the LPS, LPS-MCB, and MCB groups compared to the DMSO group, respectively. D-F) The distribution of total peaks relative to the transcription start site (TSS) in the LPS, LPS-MCB, and MCB groups, compared to the DMSO group, respectively, revealed distinct chromatin accessibility profiles in RAW 264.7 cells following 12 hours of treatment.

Supplementary Figure 7. ATAC-seq analysis reveals that MCB significantly reduces LPS-induced chromatin accessibility in RAW 264.7 cells following 12 hours of treatment. A) Heatmap showing that MCB markedly decreased LPS-induced chromatin accessibility in RAW 264.7 cells following 12 hours of treatment, with color intensity reflecting the level of accessibility. B) Heatmap illustrating the differentially accessible regions (DARs) most prominent in the LPS group compared to other groups, with color intensity representing the degree of accessibility across different experimental conditions. C) GREAT (Genomic Regions Enrichment of Annotations Tool) analysis highlighting the potential functional roles of LPS-induced DARs downregulated by MCB following 12 hours of treatment. D-E) Distribution of chromatin accessibility peaks and predicted transcription factor-binding loci relative to transcription start sites (TSS) in the LPS, LPS-MCB, and MCB groups, each compared to the DMSO control. Annotation was performed using the ChIPseeker R package.

Supplementary Figure 8. Potential transcription factors induced by LPS and MCB treatment for 12 hours in RAW 264.7 cells, as detected by ATAC-seq. A-B) Mean aggregate binding signal of transcription factors predominantly enriched in the LPS group compared to other groups, including *NFKB1*, *NFKB2*, *IRF3*, *FOSL1*, *JUND*, and *STAT1*. The data were derived from ATAC-seq footprinting analysis and revealed elevated chromatin accessibility at the corresponding binding motifs specifically in RAW 264.7 cells following 12-hour treatment, indicating enhanced transcription factor activity in response to LPS stimulation. C-D) Binding signal intensity of transcription factors predominantly enriched in the MCB group compared to the LPS group, including *CEBP1*, *SUM1*, *ARID5A*, and *SPT15*. These signals, derived from ATAC-seq footprinting analysis, indicate enhanced chromatin accessibility at their respective binding motifs, suggesting increased transcriptional regulation mediated by these factors in response to MCB treatment for 12 hours in RAW 264.7 cells.

Supplementary Figure 9. Combining ATAC-seq and RNA-seq analysis revealed coordinated changes in chromatin accessibility and gene expression in RAW 264.7 cells following a 12-hour treatment. A) Venn diagram illustrating 258 differentially expressed genes (DEGs) from RNA-seq that exhibited differentially accessible regions in the corresponding ATAC-seq analysis. B) Visualization of peak tracks for *Lta*, *Vma21*, *Acod1*, *Hmgb1*, *Atf3*, *Irf3*, *H4c9*, and *H2ac11* in the DMSO, LPS, LPS-MCB, and MCB groups using IGV software. Expression levels of differentially expressed genes (DEGs) correlated with the chromatin accessibility of *Tnf*, *Ltb*, *Lta*, *Gxylt1*, *Acod1*, *Ctla2b*, *Atf3*, *Hmgb1*, *Vma21*, and *Cxcl10* demonstrated a consistent regulatory relationship between transcriptional activity and chromatin openness in RAW 264.7 cells following LPS and MCB treatment. Statistical significance was determined using one-way ANOVA with Bonferroni’s multiple comparisons test (C). * *P* < 0.05, ** *P* < 0.01, *** *P* < 0.001 and **** *P* < 0.0001.

Supplementary Figure 10. LPS-induced *Egr1* is significantly decreased by MCB treatment. A) The expression levels of *Egr1*, *Egr2*, and *Egr3* were markedly reduced in the LPS-MCB group compared to the LPS group in RAW 264.7 cells upon 12 hours of treatment. B) The binding signal intensity of *Egr1* was stronger in the LPS group compared to the MCB group in RAW 264.7 cells upon 12 hours of treatment, as detected by the CUT&TAG. C) The mean aggregate binding signal of *Egr1* was highest in the LPS group and markedly reduced in the LPS-MCB group, with minimal signals observed in the MCB group during the 12-hour treatment. D) The binding activity of *Egr1* at the transcription start site (TSS) was substantially elevated in the LPS group, while MCB treatment significantly attenuated this activity in the LPS-MCB group. E-F) The frequency and distribution analysis of peaks uniquely expressed in response to LPS treatment, when compared to the DMSO, LPS-MCB, and MCB groups. Statistical significance was determined using one-way ANOVA with Bonferroni’s multiple comparisons test (A). * *P* < 0.05, ** *P* < 0.01, and *** *P* < 0.001.

Supplementary Figure 11. Co7 activates the innate immune response via the TLR4 receptor. A-B) Comparison of F1-score (A) and robustness to label noise (B) between VDLIN and five conventional machine learning models, including Support Vector Machine (SVM), k-Nearest Neighbors (kNN), Logistic Regression, Random Forest (RF), and Gradient Boosting (GB). Performance was evaluated on independent test sets under consistent training conditions. Robustness was assessed by introducing increasing levels of label noise (10–30%) and measuring performance degradation. C) Chemical structure and molecular weight of Co7. D) Protein-protein interaction (PPI) network analysis of differentially expressed genes (DEGs) induced by Co7 in RAW 264.7 cells compared to the DMSO group, highlighting significant enrichment in immune response pathways. E-F) Inhibition of the TLR4 signaling pathway significantly reduced Co7-induced *Ifnb1* expression in RAW 264.7 and J774 cells following 3 hours of treatment, respectively. G) Co7-induced *Ifnb1* expression was abolished in bone marrow-derived macrophages (BMDMs) and peritoneal macrophages (PMs) from TLR4 knockout (KO) mice upon 3 hours of treatment. H) Co7-induced *Ifnb1* expression was also abolished in BMDMs and PMs from TRIF KO mice upon 3 hours of treatment. RT-qPCR data were presented as means ± SEM from three independent experiments. Statistical significance was determined using one-way ANOVA followed by Bonferroni’s multiple comparisons test for panels E and F, and a paired *t*-test for panels G and H. * *P* < 0.05, ** *P* < 0.01, and *** *P* < 0.001.

Supplementary Table S1. Primer sequences used in this study.

Supplementary Table S2. Information of shRNA targeting *Egr1* used in this study.
